# Multi-targeted pharmacological actions and nanodelivery strategies of Garcinia cambogia: from molecular mechanisms to disease treatment

**DOI:** 10.3389/fchem.2025.1692386

**Published:** 2025-11-12

**Authors:** Hang Zhang, Yurou Cao, Xubin Chen, Jingxin Chen

**Affiliations:** 1 School of Stomatology, Hainan Medical University and Hainan Academy of Medical Sciences, Haikou, Hainan, China; 2 Department of Stomatology, Hainan General Hospital (Hainan Affiliated Hospital of Hainan Medical University), Haikou, China

**Keywords:** gambogic acid (GA), anti-infective effects, anti-inflammatory andantioxidant effects, anticancer effects, nanoparticle drug delivery system

## Abstract

Garcinia cambogia (Gambogic Acid, GA) is a natural xanthone compound extracted from the resin of GA fruit, renowned for its diverse biological activities and substantial therapeutic potential. GA, a principal bioactive component of Garcinia cambogia, possesses a distinctive cage-like molecular architecture centered on an α,β-unsaturated ketone moiety. This structure is not merely a chemical signature but the fundamental source of GA’s broad and integrated pharmacodynamic profile. While the multi-target nature of natural products like flavonoids has been widely documented, GA’s unique polycyclic caged structure confers a different mechanism of action and a broader spectrum of activity, particularly in epigenetic reprogramming and the activation of multi-modal cell death networks. This review moves beyond a mere compilation of GA’s effects to provide a systematic and critical analysis of its pharmacological landscape. We deconstruct its mechanisms along three integrated dimensions: (i) a molecular-level characterization of GA-regulated signaling pathways, emphasizing its multi-target synergy; (ii) an empirical evaluation of its therapeutic efficacy across cancer and inflammatory diseases, critically appraising both promises and limitations of current evidence; and (iii) an evidence-based discussion on overcoming translational barriers, with a focal point on how innovative nanodelivery strategies are pivotal in resolving GA’s pharmacokinetic challenges. By directly comparing GA with other natural products (e.g., flavonoids) in terms of structure-activity relationships and translational potential, we highlight its unique position in the natural product pharmacopeia. We conclude that the future of GA research lies in the integration of multi-omics approaches with precision drug delivery systems, a synergistic strategy that will effectively bridge the gap between its robust mechanistic underpinnings and successful clinical application.

## 1 Introduction

Garcinia cambogia (Gambogic Acid, GA) is a natural compound extracted from the resin of the *Garcinia cambogia* fruit, which is primarily found in tropical regions of Southeast Asia, Brazil, and India (Kumar et al., 2013). Historically, it has been used as a food ingredient, traditional medicine, and pigment (Guo et al., 2006; Wang et al., 2014). Gambogic acid, a naturally occurring prenylated xanthone derivative, is the most prominent member of the xanthone family, with the chemical formula C_38_H_44_O_8_ (Han et al., 2005; Hatami et al., 2020). The continuous discovery of its medicinal properties has made GA an important compound of considerable scientific and pharmacological interest. The study of GA can be traced back to its traditional medicinal applications, although systematic scientific investigation began only in the late 20th century. In Southeast Asian traditional medicine, the resin of GA was used as a laxative, anti-inflammatory agent, and topical treatment for wounds and trauma. However, its application required caution due to its potential toxicity and irritant effects. During the 19th century, chemists began isolating the active components of GA resin, but the limitations of analytical techniques at that time prevented the full elucidation of its chemical structure. It was not until the 1960s that the molecular structure of gambogic acid was identified for the first time and classified as a polyisoprenylated phenyl ketone derivative. Although its anticancer activity was recognized at that stage, its pharmacological mechanisms did not receive sufficient attention. By the early 21st century, a growing number of researchers had begun to explore and verify GA’s antitumor effects, initiating preclinical and clinical studies to assess its therapeutic potential and marking the transition of GA research from basic investigation to translational application.

GA has been shown to exert multiple pharmacological activities. Its anti-infective effects involve the inhibition of bacterial topoisomerase IV, blockage of viral spike protein binding to host cells (e.g., in hepatitis B virus infection), and regulation of the DTX1–Notch signaling pathway. Its anti-inflammatory and antioxidant properties are mediated through signaling pathways such as NF-κB and MAPK/HO-1, which contribute to the alleviation of septic organ injury and arthritis. Furthermore, GA can delay chronic disease progression by modulating pathways including HIF-1α/VEGF, thereby mitigating pathological angiogenesis and tissue hypoxia. In terms of its antitumor effects, GA has demonstrated the ability to inhibit digestive, reproductive, and hematologic cancers by inducing apoptosis, autophagy, and other forms of programmed cell death, as well as remodeling the tumor microenvironment through the regulation of noncoding RNAs and epigenetic mechanisms.

However, because GA belongs to the xanthone class of compounds characterized by a unique four-oxatricyclo [4.3.1.0 (Wang et al., 2014; Winter et al., 2013)] decan-2-one backbone, it exhibits several physicochemical limitations, including poor water solubility, low thermal stability, and weak alkali resistance (Sun et al., 2012; Winter et al., 2013). These properties have hindered its clinical translation. Nevertheless, the study by Arevalo et al. (2022) reported an efficient isolation method capable of purifying GA from commercially available GA resin with a diastereomeric purity greater than 97%, at a production cost lower than that of synthetic drugs. Although numerous studies have confirmed GA’s diverse pharmacological effects as a naturally active compound (Li et al., 2022a), further research is still needed to address and overcome the inherent structural limitations that restrict its practical application (Tu et al., 2024). These challenges can be addressed by structural modification of GA to enhance its bioavailability (Ke et al., 2022; Na et al., 2020; Wang et al., 2024; Zhang Y. et al., 2024). At present, drug resistance remains a major obstacle in cancer therapy; however, as discussed later in this paper, GA has demonstrated promising efficacy against chemotherapy-resistant tumors, particularly triple-negative breast cancer. Moreover, in the context of certain chronic inflammatory diseases, GA may serve as a safer alternative to conventional anti-inflammatory agents such as glucocorticoids, which are associated with significant adverse effects when used long term. In terms of clinical research progress, China has approved a liposomal formulation of GA for phase II clinical trials (NCT04386915). Preliminary findings have shown a 50% reduction in systemic toxicity and greater therapeutic efficacy compared with free GA. Nonetheless, a multicenter phase III clinical trial is still needed to further evaluate the first-line therapeutic potential of GA nanoformulations in solid tumors. Recent studies have also reported that an injectable nanocomposite hydrogel loaded with curcumin can deliver drugs in a targeted and controlled manner through minimally invasive *in situ* injection. By remodeling the immunosuppressive tumor microenvironment, this delivery system enhances antitumor efficacy (Lei et al., 2025). Integrating the multi-target pharmacological properties of gambogic acid with nanotechnology-based delivery systems is expected to overcome current therapeutic bottlenecks. The clinical translation of such strategies holds not only significant scientific importance but also the potential to provide cost-effective therapeutic options for patients worldwide.

A critical factor in drug translation is the evaluation of systemic toxicity. Assessing GA’s toxicity profile in the broader context of plant-derived anticancer agents enables a more comprehensive understanding of its pharmacological characteristics. Natural anticancer compounds such as camptothecin and paclitaxel—both well known in traditional Chinese medicine—are effective but also recognized for their inherent toxicity (Wang et al., 2012). Compared with widely used clinical chemotherapeutic drugs, GA exhibits lower bone marrow toxicity (Chi et al., 2013). In contrast, when compared with curcumin, another multi-target natural compound known for its remarkably low bioavailability and exceptional safety profile (Gupta et al., 2013), GA demonstrates stronger *in vitro* antitumor activity but also higher systemic toxicity. Its severe *in vivo* toxicity and poor pharmacokinetic behavior remain major barriers to clinical translation.

Although sporadic studies have reported formulation optimizations to improve GA’s pharmacological performance, there is still a lack of comprehensive review and critical evaluation of advanced delivery strategies, particularly nanotechnology-based targeted systems. Furthermore, the synergistic potential of GA in combination with conventional chemotherapeutic agents or immune checkpoint inhibitors—a rapidly developing research area—has not yet received sufficient attention in previous reviews. Therefore, this paper builds upon and expands current understanding of GA’s pharmacological mechanisms by systematically elucidating the design principles and research progress of diverse nanocarrier systems developed to enhance the drugability of gambogic acid. It also explores novel combination therapy strategies and their underlying mechanisms. We anticipate that this review will provide practical insights to advance GA from fundamental research toward clinical application.

## 2 The Chemical foundation of gambogic acid

As briefly introduced previously, GA is the principal active component extracted from Garcinia resin, belonging to the class of caged tetracyclic xanthonoids. Its chemical structure is defined by the following core characteristics.

### 2.1 Molecular backbone

**FIGURE 1:**
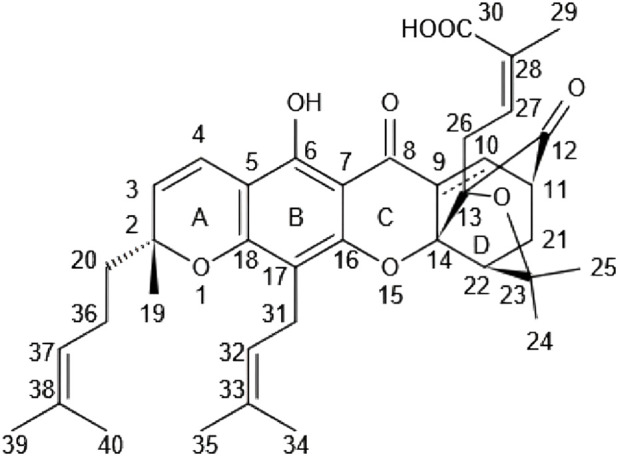
Chemical structure of GA (reprinted from Li et al., 2022a, CC BY).

As illustrated in Figure 1, GA features a polycyclic anthraquinone-like core, specifically a tetracyclic xanthonoid structure comprising four fused carbon rings. This forms an extensive hydrophobic region, which is the primary reason for its strong lipophilicity and extremely low water solubility. Furthermore, its molecular formula is C_38_H_44_O_8_ (molecular weight ≈ 628.75 g/mol); the large molecular weight and complex structure further restrict its aqueous solubility (Na et al., 2020).

### 2.2 Key functional groups: determinants of chemical properties and biological functions

The complex and unique bioactivity of GA does not stem from a single functional group but from the synergy and balance among several key moieties. Collectively, these groups define the molecule’s overall physicochemical properties (e.g., solubility, stability) and biological interactions (e.g., target binding, prodrug design). The following sections deconstruct how these core functional groups define GA’s “chemical personality” from different dimensions, ultimately laying the foundation for its pharmacological actions and nanodelivery strategies.

Firstly, the physicochemical properties and specific delivery strategies of the molecule are primarily determined by its carboxylic acid and ester linkage. The terminal carboxylic acid group imparts weak acidity, allowing GA to form complexes with metal ions like Ca^2+^ (e.g., Ca^2+^-GA carboxylate). This is the key mechanism enabling drug loading into the aqueous interior of liposomes via solvent-assisted active loading (SALT). The polarity of this group also enhances solubility in organic solvents like ethanol, facilitating entry into liposomes. However, due to the dominance of the polycyclic hydrophobic structure, overall water solubility remains extremely low (<0.0050 mg/mL) (Na et al., 2020). On the other hand, the ester linkage serves as an ideal site for constructing hydrophobic prodrugs. For instance, the prodrug formed by connecting GA to oleyl alcohol via this bond is stable in pH 7.4 PBS but is selectively hydrolyzed in esterase-rich environments like plasma or the tumor microenvironment, enabling targeted release (Wang et al., 2024).

Secondly, the phenolic hydroxyl at C6 and the chiral center at C2 are critical markers for analytical identification and stability characterization. The strongly polar phenolic hydroxyl at C6 appears at a chemical shift of 12.75 ppm (12.76 ppm for epi-GA) in ^1^H NMR using CDCl_3_, serving as a key signature to distinguish GA from its C2 epimer and participating in the formation of the ortho-quinone methide intermediate (Arevalo et al., 2022). The absolute configuration at the C2 chiral center is R, but it readily undergoes epimerization to the S-configured epi-gambogic acid. This epimer is a major contaminant in commercial GA samples and is central to the molecule’s stereochemical instability (Arevalo et al., 2022).

Thirdly, the molecule’s lipophilicity, reactivity, and core pharmacophore constitute the cornerstone of its pharmacological action. Multiple prenyl side chains significantly enhance GA’s lipophilicity and promote hydrophobic interactions with biological membranes or target proteins. These are hallmark substituents of prenylated xanthones and are likely involved in cell membrane penetration and target binding (Ke et al., 2022). However, the most prominent pharmacophore is the reactive α,β-unsaturated ketone group located within the caged CD ring structure. This group acts as a strong electrophilic center, capable of covalently modifying free thiol groups in intracellular proteins via Michael addition reactions. This interferes with disulfide bond formation, leads to the accumulation of misfolded proteins, and induces cell vacuolation and death. Furthermore, this covalent interaction disrupts protein folding, inducing endoplasmic reticulum and mitochondrial stress, ultimately causing cancer cell death. This structural region (particularly the BC plane) is essential for its antitumor activity and serves as a key site for interactions with targets (e.g., the proteasome, NF-κB pathway proteins) via hydrogen bonding or electrophilic interactions (Na et al., 2020).

Finally, the susceptibility of multiple functional groups, including ketone and ester groups, to chemical degradation dictates that GA must be stored properly. These groups are prone to oxidation or hydrolysis, accounting for GA’s sensitivity to light and heat. Consequently, storage away from light at low temperatures (4 °C) is required, with most reports indicating stability can be maintained for 30–45 days under these conditions (Ke et al., 2022; Na et al., 2020).

Understanding this chemical profile is paramount. It explains the imperative for advanced delivery systems to solubilize GA, shield its reactive sites to reduce premature reactions and systemic toxicity, and guide it precisely to the desired therapeutic targets.

The GA molecule contains multiple modifiable sites (e.g., the C-30 carboxyl, C-9/10 double bond, C-34/39 allylic positions), allowing covalent conjugation via reactions like esterification, amidation, or epoxidation with polymers or targeting molecules to form amphiphilic prodrug nanomicelles. Furthermore, the α,β-unsaturated ketone structure acts as an electrophilic center, enabling stable drug-carrier conjugation via Michael addition reactions with thiol-containing proteins or polymers (Li et al., 2022a).

The specific functional groups in GA’s structure, such as hydroxyl and ketone groups, can undergo chemical reactions (e.g., esterification, amidation) to covalently link with polymers (e.g., PEG, PLGA) or targeting molecules (e.g., folic acid, hyaluronic acid), forming stable nano-conjugates. Its chemical structure also allows for triggered release under specific conditions (e.g., acidic pH, high glutathione concentration). The low water solubility, inherent due to the fused carbon rings, facilitates its encapsulation within hydrophobic cores (e.g., of PLGA nanoparticles, liposomes), preventing premature degradation in the bloodstream. In essence, GA’s chemical reactivity facilitates functionalization, while its hydrophobicity and complex stability support the longevity and targeting capabilities of nano-delivery systems, thereby overcoming the limitations for clinical application (such as low bioavailability and non-specific toxicity) (Fahmy et al., 2024).

Therefore, GA’s chemical functions are highly integrated: its carboxylic acid provides an anchor for molecular modification and specific delivery strategies; its phenolic hydroxyl and chiral center impact its analytical identification and stability; its prenyl groups and fused carbon rings collectively create a strongly hydrophobic core; and all these structures ultimately support the powerful electrophilic reactivity of its core pharmacophore—the α,β-unsaturated ketone. This reactivity is both the powerful driver behind GA’s multi-targeting pharmacology and the root of its off-target toxicity. This inherently explains the imperative for nanodelivery systems to “harness the strengths and circumvent the weaknesses”—by utilizing its modifiability for encapsulation or targeting design while shielding its reactivity until precise delivery to the target site is achieved.

## 3 Toxicity profile and safety considerations of gambogic acid

Although numerous studies have confirmed the broad-spectrum pharmacological activities demonstrated by GA, its significant systemic toxicity and poor pharmacokinetic properties hinder its translational potential. A comprehensive understanding of its safety profile is primarily derived from *in vivo* studies.

### 3.1 Systemic toxicity in animal models

In rodent models, the maximum tolerated dose (MTD) of GA via intravenous administration is reported to be around 50–60 mg/kg, with an LD_50_ of approximately 40 mg/kg (Guo et al., 2006; Tu et al., 2024). The primary dose-limiting toxicities include hepatotoxicity and cardiotoxicity, manifesting as lethargy, reduced activity, and at higher doses, acute organ failure.

### 3.2 Hematological effects

A notable feature of GA is its relatively lower bone marrow toxicity compared to conventional chemotherapeutics like paclitaxel or cisplatin (Chi et al., 2013). Clinical phase IIa trials reported that GA caused grade 3/4 neutropenia in only 6.25% of patients, a rate significantly lower than that typically associated with many standard chemotherapy regimens (Chi et al., 2013).

### 3.3 Organ-specific toxicities

(i) Hepatotoxicity: The liver is a primary target for GA-induced toxicity. Studies in mice show that GA administration leads to a significant increase in serum alanine aminotransferase (ALT) and aspartate aminotransferase (AST) levels, indicating hepatocellular injury (Guo et al., 2006; Tu et al., 2024). Histopathological examination reveals hepatic sinusoidal obstruction syndrome (SOS) and hemorrhagic necrosis. (ii) Cardiotoxicity: GA can induce arrhythmia and a decrease in myocardial contractility in dogs and rodents, potentially linked to its inhibition of hERG potassium channels (Tu et al., 2024). (iii) Nephrotoxicity: Although less prominent than hepatotoxicity, mild to moderate renal toxicity is observed, evidenced by elevations in blood urea nitrogen (BUN) and creatinine (CRE) levels in animal models (Li et al., 2023).

### 3.4 Comparative toxicity with other plant-derived compounds

(i) Compared to Camptothecin and Paclitaxel: GA exhibits a different toxicity spectrum. While camptothecin and paclitaxel are notorious for severe myelosuppression and neurotoxicity, GA’s primary challenge is hepatotoxicity. However, its lower incidence of myelosuppression could be an advantage in combination therapies (Chi et al., 2013; Wang et al., 2012). (ii) Compared to Curcumin: Curcumin is celebrated for its exceptional safety profile but suffers from extremely low bioavailability (Gupta et al., 2013). In contrast, GA possesses superior *in vitro* potency but carries a much higher risk of systemic toxicity, underscoring the critical need for targeted delivery systems to dissociate its efficacy from its toxicity.

In conclusion, the development of nano-formulations, as discussed in subsequent sections, is primarily motivated by the imperative to mitigate these well-documented toxicities while enhancing the therapeutic index of GA.

## 4 Garcinia cambogia pharmacological mechanism of action

The following sections will dissect the multifaceted pharmacology of GA. Rather than a catalogue of disjointed effects, we present a cohesive narrative of how GA, from its unique chemical foundation, mounts a multi-front assault on the common soil of disease—oxidative stress and chronic inflammation. This journey will begin with its direct anti-infective actions, then reveal how its potent anti-inflammatory and antioxidant capabilities not only resolve acute damage but also intervene in the progression of chronic diseases. Ultimately, we will demonstrate how these foundational mechanisms converge and amplify in its anti-cancer campaign, through epigenetic reprogramming, activation of a multi-modal cell death network, and systemic remodeling of the tumor microenvironment. Throughout this narrative, the theme of GA’s multi-target nature will emerge as both its greatest strength and its primary translational challenge—a challenge that logically culminates in the discussion of innovative nanodelivery strategies designed to harness this complex pharmacology for clinical benefit.

### 4.1 Anti-infective effects

GA has demonstrated remarkable potential in anti-infective therapy. Recent studies have revealed that GA combats drug-resistant pathogens through a multitarget antimicrobial mechanism, making it a promising candidate for addressing the escalating global challenge of antimicrobial resistance. While global collaboration remains crucial in combating resistance, natural products such as GA—especially when integrated with emerging technologies like artificial intelligence–assisted drug design and nanotechnology-based delivery systems—may provide innovative solutions that transcend the limitations of traditional antibiotics.

#### 4.1.1 GA and gram-positive bacteria

Garcinia cambogia primarily exerts its antimicrobial activity against Gram-positive bacteria, particularly *Staphylococcus aureus* (including methicillin-resistant strains, MRSA) (Chaiyakunvat et al., 2016) and *Enterococcus* species (including vancomycin-resistant enterococci, VRE) (Li et al., 2022b). GA exhibits strong antimicrobial activity by inhibiting bacterial cell wall synthesis through targeting *Enterococcus faecalis* undecaprenyl pyrophosphate synthase (EfaUPPS) (Li et al., 2022b). Comprehensive *in vivo* and *in vitro* studies have confirmed that GA inhibits bacterial cell wall synthesis by binding to the EfaUPPS enzyme, thereby disrupting the biosynthesis of undecaprenyl pyrophosphate (UPP), an essential bacterial cell wall precursor. Mechanistically, GA occupies the substrate-binding pocket of EfaUPPS—competing with farnesyl pyrophosphate (FPP)—thereby interrupting peptidoglycan biosynthesis and ultimately leading to bacterial cell death. The microtiter broth dilution method revealed a minimum inhibitory concentration (MIC) of GA against *E. faecalis* of 2 μg/mL. Enzyme activity analysis using fluorescence detection yielded an IC_50_ value of 3.08 μM. Subsequent mouse infection studies demonstrated that treatment with 40 μM GA significantly alleviated E. faecalis–induced splenomegaly (p < 0.01). Although the study compared GA’s *in vitro* antibacterial activity with selected frontline clinical antimicrobials—primarily validating its efficacy through positive control drug experiments—it did not comprehensively evaluate its comparative efficacy against a broader range of clinically used antibiotics.

Against enterococci, particularly VRE strains, GA interferes with bacterial DNA replication by binding to the ParE subunit of topoisomerase IV and inhibiting its ATPase activity, thereby blocking bacterial cell division and replication. When used as an antimicrobial adjuvant, GA markedly enhances the antibacterial effect of vancomycin against VRE (Pang et al., 2024). *In vitro* experiments demonstrated significant differences between GA used alone and standard antibiotics such as vancomycin (VAN) or ampicillin (AMP) used alone: VAN exhibited MICs ≥32 μg/mL and AMP >256 μg/mL, indicating strong resistance, whereas GA showed MICs of only 2–4 μg/mL. Co-administration of GA with vancomycin reduced the latter’s MIC by 4- to 1024-fold, as indicated by the Drug Reduction Index (DRI). Additionally, GA displayed an IC_50_ value of 6.96 μg/mL, while neomycin’s IC_50_ against enterococci was 5.89 μg/mL, demonstrating that GA performs comparably or even more effectively than conventional antibiotics in both MIC and target inhibition activity. Importantly, GA’s unique mechanism of action—targeting ParE—has the potential to circumvent existing antibiotic resistance, providing a novel strategy for clinical antimicrobial therapy. *In vivo* experiments using a mouse multi-organ infection model further confirmed that GA, when combined with vancomycin, produced significant synergistic antibacterial effects, particularly in the liver and lungs, and markedly reduced bacterial load in infected tissues.

#### 4.1.2 GA and chikungunya virus

Chikungunya fever (CHIK) is a mosquito-borne viral disease caused by the Chikungunya virus (CHIKV), for which no effective antiviral drug or vaccine is currently available (Schuffenecker et al., 2006). Indian researchers have proposed that the mechanism by which GA acts against CHIKV infection is primarily revealed through molecular docking analyses, focusing on its interaction with the viral envelope glycoprotein E2. GA has been shown to bind to several key amino acid residues within the active site of the E2 protein through hydrogen bonding and hydrophobic interactions. These interactions may interfere with the binding of E2 to its host receptor, MXRA8, thereby preventing viral entry into host cells. Computational modeling thus suggests that GA may serve as a potential anti-CHIKV candidate compound (Qamar et al., 2023). However, current studies investigating the mechanism of GA against CHIKV rely mainly on molecular docking results and lack sufficient experimental validation. The evidence remains preliminary and limited in robustness, as it is based on a single source of *in silico* data. Therefore, these findings require systematic confirmation through subsequent cell-based and biochemical *in vitro* experiments before firm conclusions can be drawn regarding GA’s antiviral efficacy and mechanism of action.

#### 4.1.3 GA and the HBV virus

Hepatitis B virus (HBV) infection remains a major global public health challenge. Despite the availability of effective vaccines and antiviral therapies, chronic hepatitis B (CHB) continues to be a leading cause of liver failure, cirrhosis, and hepatocellular carcinoma (HCC) (Global et al., 2022). Researchers initially identified GA as a promising anti-HBV compound after screening 715 traditional Chinese herbal medicines. MTT assays verified its low cytotoxicity, while qRT-PCR and Western blot analyses confirmed that GA inhibited the expression of Flag-HBx mRNA and protein in a dose-dependent manner. Further experiments demonstrated that GA or DTX1 overexpression upregulated key genes in the Notch signaling pathway, such as Notch1 and Hes1, whereas DTX1 knockout suppressed this pathway. These results indicate that GA inhibits HBV replication mediated by the HBx protein through activation of the DTX1–Notch signaling pathway. The DTX1 gene plays a critical role in regulating HBV replication and translation, and activation of the Notch pathway appears to be central to GA’s anti-HBV effect (Wen et al., 2024). In a mouse model of HBV cccDNA infection established via hydrodynamic tail vein injection, treatment with GA (2.5 mg/kg) significantly reduced serum HBV DNA levels, HBeAg/HBsAg expression, and hepatic HBV RNA/DNA levels, achieving therapeutic effects comparable to those of the positive control drug entecavir (ETV). Collectively, these multidimensional experiments confirm that GA, as a natural bioactive compound, significantly suppresses HBx protein–mediated HBV replication by modulating the DTX1–Notch signaling pathway, providing new mechanistic insights and potential therapeutic strategies for chronic HBV infection (Wen et al., 2024).

**FIGURE 2:**
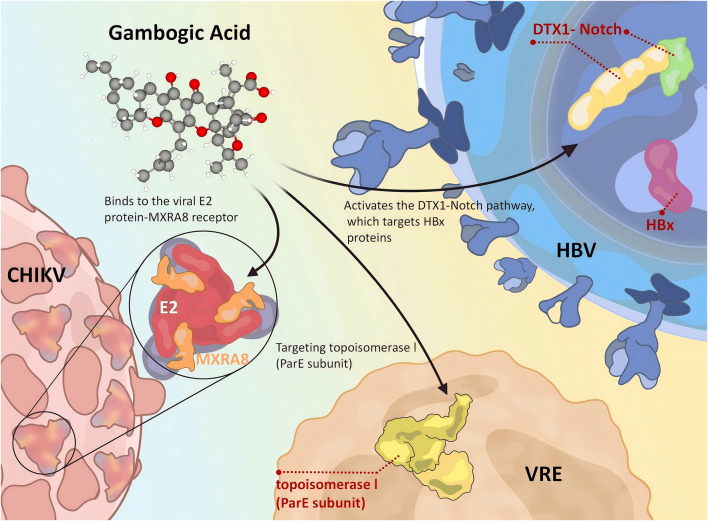
Mechanism of GA anti-infection.

In summary, GA exhibits multi-targeted anti-infective potential by inhibiting bacterial topoisomerase IV, blocking viral entry, and modulating host signaling pathways, with its core mechanisms summarized in Figure 2. GA not only directly eliminates pathogens but also strategically modulates the host’s defensive response. This dual approach—targeting both the invader and the host’s potentially detrimental inflammatory reaction—seamlessly introduces its next, and perhaps more profound, pharmacological dimension: its potent capacity as a broad-spectrum anti-inflammatory and antioxidant agent, which we will now explore.

### 4.2 Anti-inflammatory and antioxidant effects

**FIGURE 3:**
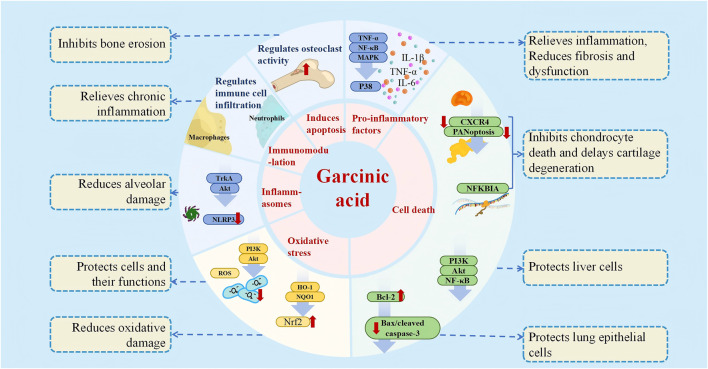
Anti-inflammatory and antioxidant mechanism of GA.

As foreshadowed by its role in tempering infection-induced inflammation, GA’s anti-inflammatory and antioxidant properties represent a therapeutic expansion from combating external threats to resolving internal dysregulation. This section will detail how GA, by quenching the dual flames of oxidative stress and inflammation, intervenes in a wide array of diseases where these processes are the common soil. The anti-inflammatory and antioxidant effects of GA, which form the cornerstone for treating multiple diseases, are mediated through the regulation of key signaling pathways such as NF-κB, MAPK, and Nrf2 across various models, as illustrated in Figure 3 and detailed for specific diseases in Table 1.

**TABLE 1:** Pharmacological effects of GA in ocular chronic diseases.

Disease	Core mechanism	Observed therapeutic effect	Refs
Diabetic retinopathy (DR)	(1) Inhibits the HIF-1α/VEGF signaling axis, reducing pathological angiogenesis (2) Activates the Nrf2 pathway, upregulating HO-1/NQO1 to alleviate oxidative stress and inflammation	Inhibits retinal neovascularization, alleviates inflammatory damage, improves retinal structural abnormalities	Chen et al. (2021), Cui et al. (2018)
Diabetic cataract (DC)	(1) Binds to hydrophobic sites on γ-crystallin, inhibiting heat-induced protein aggregation (2) Mimics the function of α-crystallin chaperone, maintaining protein stability	Delays lens clouding, maintains lens transparency	Islam et al. (2022)

This table summarizes the primary pharmacological mechanisms and therapeutic effects of Gambogic Acid (GA) in major ocular chronic diseases. The actions are categorized by disease pathology.

Notably, the anti-infective effects of GA extend beyond direct pathogen clearance. GA can effectively suppress infection-induced hyperinflammatory responses by modulating the host immune system. It exhibits disease-modifying properties through multiple mechanisms, including but not limited to: (i) inhibition of pro-inflammatory cascade responses through modulation of [specific pathways]; (ii) scavenging of reactive oxygen species to mitigate oxidative stress; and (iii) exertion of coordinated multi-organ protective effects through the synergistic regulation of diverse molecular targets. These multifaceted pharmacological actions highlight GA’s broad therapeutic potential across a wide range of inflammatory and oxidative stress-related diseases.

#### 4.2.1 GA and arthritis

Rheumatoid arthritis (RA) is a chronic autoimmune inflammatory disease (Smolen et al., 2016). Non-steroidal anti-inflammatory drugs (NSAIDs) and corticosteroids remain the mainstay treatments for RA (Di Matteo et al., 2023; Katz et al., 2021); however, these therapies primarily alleviate symptoms rather than address the underlying pathology, and they are often associated with significant systemic side effects (Arfeen et al., 2024). Moreover, their therapeutic efficacy is further constrained by poor targeted drug delivery, which limits the concentration of active agents in inflamed tissues. To overcome these challenges, researchers have developed polymer-based nano-delivery systems. Nanomedicines can be passively enriched in chronic inflammatory tissues through the “ELVIS effect,” namely extravasation through leaky vasculature and inflammatory cell-mediated sequestration (Xin Li et al., 2023). Leveraging the potent anti-inflammatory and antioxidant properties of curcumin, Liu Y. et al. developed nanoparticles incorporating GA as a bioactive component (GBA2/NPs). In extensive *in vitro* and *in vivo* studies, GBA2/NPs demonstrated superior cytotoxicity, cellular uptake, and pro-apoptotic activity in osteoblasts and macrophages compared with free GA. *In vivo*, GBA2/NPs accumulated more effectively at inflamed joint sites, significantly alleviated arthritic symptoms, protected cartilage tissue, and reduced inflammation, while exhibiting markedly lower systemic toxicity than free GA (Liu Y. et al., 2023). Similarly, Nguyen A. synthesized self-assembled nanoparticles (NPs) by chemically coupling short-chain methoxy polyethylene glycol (mPEG) with GA—a small-molecule anti-inflammatory compound—using a click chemistry platform. By optimizing the polymer–drug conjugate composition and physicochemical properties, the performance of this drug delivery system was substantially enhanced. Mechanistically, the anti-arthritic effect of GA is mainly attributed to its anti-inflammatory activity through inhibition of inflammatory mediators and suppression of the NF-κB/TNF-α signaling pathway (Nguyen et al., 2020). Collectively, these findings suggest that synthetic nanoparticle technology offers a promising therapeutic approach for RA treatment.

Osteoarthritis (OA) is another highly prevalent orthopedic disorder characterized by progressive degeneration of articular cartilage. Its exact pathogenesis remains unclear, and treatment is largely limited to symptomatic management (Felson, 2006). NSAIDs and glucocorticoids can relieve pain and inflammation but are associated with notable adverse effects, while joint replacement surgery is costly and unsuitable for most patients (Krasnokutsky et al., 2007). Using bioinformatics analysis, Zhang Y. Y. identified PANoptosis-related biomarkers—representing an integrated form of programmed cell death combining pyroptosis, apoptosis, and necroptosis—in OA. Drug prediction based on NFKBIA analysis identified 12 potential therapeutic compounds, including GA. The study found that GA may slow OA progression by downregulating chemokine receptor 4 (CXCR4) expression in chondrocytes and reducing PANoptosis-related activity (e.g., NFKBIA), suggesting a potential novel therapeutic mechanism for OA (Zhang Y. Y. et al., 2023). However, since these findings are derived solely from bioinformatics and pharmacological prediction analyses, further *in vitro* and *in vivo* validation is required.

Compared with standard therapies, curcumin- or GA-based nanoparticles exhibit several notable advantages. Mechanistically, they achieve multi-target anti-inflammatory effects by regulating key signaling pathways such as NF-κB, rather than relying solely on COX inhibition as NSAIDs do. In addition, they demonstrate potential chondroprotective effects. From a safety perspective, nanotechnology-enabled targeted delivery allows efficient accumulation within inflamed joints, potentially reducing systemic exposure and adverse reactions. Nevertheless, additional animal studies are necessary to confirm whether their systemic toxicity is indeed lower than that of equivalently effective NSAIDs or corticosteroids.

#### 4.2.2 GA and liver injury

Because drugs and nutrients are metabolized primarily in the liver after absorption, this organ is highly vulnerable to injury during the biotransformation of exogenous substances (Zheng et al., 2018). Overuse of over-the-counter (OTC) analgesics, particularly acetaminophen (APAP), remains the most common pharmacological cause of hepatotoxicity (Elshamy et al., 2021; Kuznietsova et al., 2019). Oxidative stress and inflammation are recognized as the core mechanisms underlying APAP-induced liver injury (Ge et al., 2019). Numerous studies have demonstrated that natural antioxidant compounds can effectively prevent or mitigate both acute and chronic hepatotoxicity. In a rat model of APAP-induced acute liver injury, Ding Z. and colleagues found that GA exerted potent hepatoprotective effects through multiple mechanisms. These included: antioxidant effects: ↓MDA (malondialdehyde), ↑SOD (superoxide dismutase)/CAT (catalase)/GPx (glutathione peroxidase)/GST (glutathione-S- transferase), ↓4-HNE (4-hydroxynonenal); anti-apoptotic effects: ↓Bax (pro-apoptotic protein)/caspase-3/9 (apoptosis executing protease), ↑Bcl-2 (anti-apoptotic protein), activation of the PI3K/Akt pathway; anti-inflammatory effects: ↓TNF-alpha (tumor necrosis factor-alpha)/IL-1beta (interleukin-1beta)/IL-6 (interleukin-6), ↓p-NF-κB (phosphorylated nuclear factor κB), ↓PGE2 (prostaglandin E2); functional recovery: ↓AST (aspartate aminotransferase)/ALT (alanine aminotransferase)/ALP (alkaline phosphatase), and amelioration of histopathological damage. Collectively, these findings indicate that GA protects against acute hepatotoxicity primarily by regulating the PI3K/Akt/NF-κB signaling pathway, suppressing oxidative stress, and activating anti-apoptotic responses (Ding et al., 2021). These multidimensional protective mechanisms highlight GA’s therapeutic potential as a natural hepatoprotective agent.

#### 4.2.3 GA and sepsis-related myocardial injury

Sepsis-associated myocardial injury (SMI) is a frequent and severe complication of sepsis, typically presenting as reversible myocardial depression (L'Heureux et al., 2020). Its pathogenesis is multifactorial, involving myocardial fibrosis, mitochondrial dysfunction, apoptotic damage, autophagy dysregulation, disturbances in autonomic control, calcium-handling abnormalities, oxidative stress, and inflammatory responses (Kakihana et al., 2016). Fu W. et al. established a mouse model of sepsis by intraperitoneal injection of lipopolysaccharide (LPS) in male C57BL/6 mice, followed by treatment with various doses of GA or vehicle (DMSO). The results demonstrated that GA exerted significant anti-apoptotic, anti-fibrotic, and anti-inflammatory effects by inhibiting the p38 MAPK/NF-κB signaling pathway, thereby effectively mitigating LPS-induced sepsis-related myocardial injury and improving cardiac function (Fu et al., 2022). Mechanistically, GA dose-dependently improved LPS-induced cardiac dysfunction through: (i) decreasing serum and tissue markers of cardiac injury; (ii) restoring hemodynamic stability; (iii) inhibiting cardiomyocyte apoptosis; (iv) attenuating myocardial fibrosis; and (v) suppressing p38 MAPK/NF-κB-mediated inflammatory signaling. Given the high morbidity and mortality associated with sepsis-related myocardial injury, these findings suggest that GA may serve as a promising therapeutic candidate. Further studies—particularly clinical and translational investigations—are warranted to clarify its mechanisms of action and evaluate its potential for clinical application.

#### 4.2.4 GA and kidney injury

Acute kidney injury (AKI) is a common clinical syndrome (Ronco et al., 2019), and its pathogenesis mainly involves oxidative stress and inflammatory response (Pickkers et al., 2021), and effective pharmacological treatments remain limited beyond supportive therapy (Chen et al., 2023). Since China’s Drug Administration approved GA for phase II clinical trials in cancer treatment, it has demonstrated good safety and tolerability while exerting antioxidant and anti-inflammatory effects via activation of the Nrf2 signaling pathway and inhibition of the NF-κB pathway. However, GA’s poor water solubility limits its therapeutic efficacy in AKI. To overcome this limitation, researchers have developed GA nanoparticles (GA-NPs) to enhance renal targeting and improve therapeutic outcomes. Studies have shown that GA-NPs exhibit higher renal retention and efficacy in AKI mice. PET imaging confirmed that the retention time of Al^18^F-GA-NPs in AKI mice was significantly longer than in healthy mice, indicating increased renal uptake. Laboratory analyses demonstrated that GA-NPs reduced oxidative stress, significantly decreased creatinine (CRE) and blood urea nitrogen (BUN) levels, and improved renal function. Safety evaluations in healthy mice showed no significant changes in body weight or blood markers, confirming the biocompatibility of GA-NPs. By improving renal uptake, GA-NPs exhibited pronounced renoprotective effects with excellent biosafety, highlighting their potential as a novel therapeutic strategy for AKI (Li et al., 2023).

#### 4.2.5 GA and inflammatory skin diseases

The pathogenesis of inflammatory skin diseases—such as atopic dermatitis, acne, and psoriasis—and skin cancers often involves oxidative stress combined with chronic inflammatory responses (Pleguezuelos-Villa et al., 2020). Xanthones, such as those found in GA, can modulate pro-inflammatory cytokines (e.g., IL-1β, IL-6, IL-8, TNF-α) and anti-inflammatory cytokines (e.g., IL-10), and influence immune cell recruitment, activation, and infiltration through the NF-κB and MAPK signaling pathways. Given its multitarget pharmacological activity, GA is considered a promising therapeutic agent for inflammatory skin diseases due to its antioxidant, anti-inflammatory, and anti-tumor effects (Gunter et al., 2020). However, the detailed molecular mechanisms remain incompletely understood. Future research should focus on elucidating the structure–activity relationships, molecular mechanisms, and therapeutic applications of GA in inflammatory skin disorders.

#### 4.2.6 GA and neonatal pneumonia

Neonatal pneumonia (NP) is a severe infectious disease of the respiratory system, associated with high morbidity and mortality (Duke, 2005; Nissen, 2007). Misuse of antibiotics has led to increasing drug resistance (Jin et al., 2015; Voulgari et al., 2014), highlighting the urgent need for novel therapeutic strategies. In an LPS-induced neonatal pneumonia cell model, GA was shown to attenuate inflammatory and apoptotic injury via the TrkA/Akt signaling pathway (Gao et al., 2021). Low to moderate concentrations of GA (1–100 nM) significantly protected WI-38 cells from LPS-induced cell death, whereas higher concentrations (≥100 nM) caused cytotoxicity. GA pretreatment markedly reduced LPS-induced apoptosis and inhibited the production of inflammatory proteins IL-6 and MCP-1. Western blot analysis further demonstrated that GA enhanced TrkA phosphorylation and Akt activation in LPS-treated cells. Notably, Akt knockdown significantly diminished the protective effects of GA, indicating that Akt signaling is central to GA’s cytoprotective mechanism. Although these findings are based on cellular experiments, they suggest GA’s potential therapeutic value in preventing NP-associated cellular injury. These results provide a molecular basis for further studies, supporting the development of novel therapeutic strategies for neonatal pneumonia. Future validation through animal studies and clinical trials is required.

As evidenced across these diverse models of acute inflammation and oxidative injury, GA functions as a potent modulator of the body’s defense systems. However, the true therapeutic significance of this capacity is fully realized when we consider that chronic, low-grade inflammation and persistent oxidative stress are the common pathogenic soil from which a vast spectrum of non-communicable chronic diseases arise. The ability of GA to systemically quench these processes, therefore, positions it not merely as a remedy for acute damage, but as a strategic agent capable of intervening in the core pathophysiology of conditions like diabetes complications, degenerative disorders, and fibrotic diseases, which we will explore next.

### 4.3 GA and chronic diseases

Building upon GA’s established role in counteracting acute inflammatory and oxidative insults, we now turn to its implications in chronic diseases. The mechanisms detailed previously—such as the inhibition of NF-κB, activation of Nrf2, and modulation of MAPK pathways—are not confined to resolving transient threats. Instead, they form the foundational arsenal through which GA addresses the sustained cellular dysfunction that characterizes conditions like diabetic retinopathy, polycystic kidney disease, and systemic fibrosis. This section will delineate how GA’s targeting of shared pathological hubs translates into therapeutic benefits across disparate chronic conditions, thereby exemplifying its capacity for systemic, mechanism-based intervention.

**FIGURE 4:**
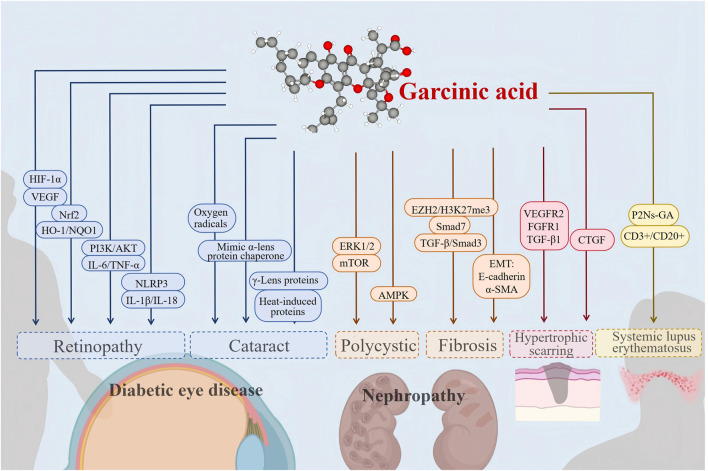
Effectiveness and mechanism of GA action on chronic diseases.

Numerous studies have demonstrated that chronic low-grade inflammation and oxidative stress imbalance are key drivers of metabolic syndrome, atherosclerosis, neurodegenerative diseases, and other conditions. For instance, in type 2 diabetes, macrophage infiltration into adipose tissue triggers an inflammatory response that contributes to insulin resistance (Lee and Lee, 2014). In Alzheimer’s disease, β-amyloid deposition activates microglia and generates excessive ROS, accelerating neuronal damage (Bai et al., 2022). GA may provide a theoretical basis for the treatment of chronic diseases by modulating these common mechanisms through multiple targets (Figure 4) (Table 1).

#### 4.3.1 GA and eye disease

Diabetic retinopathy (DR) is a major microvascular complication of diabetes, which can lead to vision loss and even blindness (Yau et al., 2012). Its core pathological mechanism involves hypoxia-induced activation of the HIF-1α/VEGF pathway, promoting retinal neovascularization (Voiculescu et al., 2015; Wert et al., 2016; Wu et al., 2015). To investigate GA’s efficacy and mechanism in DR, Cui, J. et al. demonstrated that GA dose-dependently inhibited high-glucose-induced retinal endothelial cell proliferation, migration, and tube formation *in vitro*, and improved retinal structural abnormalities while reducing neovascularization in diabetic mice *in vivo*. These effects were achieved by inhibiting the HIF-1α/VEGF signaling axis and modulating the network of pro-angiogenic factors. Furthermore, GA inhibited the PI3K/AKT pathway, and activation of this pathway by IGF-1 could reverse GA’s anti-angiogenic effect, highlighting GA’s central role in this pathway and its potential as an anti-angiogenic therapeutic strategy for DR (Cui et al., 2018). In addition to these pathways, GA exerts anti-inflammatory effects through activation of the Nrf2 (nuclear factor E2-related factor-2) signaling pathway, promoting Nrf2 nuclear translocation and upregulating downstream antioxidant genes, including HO-1 (heme oxygenase-1) and NQO1 (NAD(P)H: quinone oxidoreductase 1), thereby inhibiting activation of the NLRP3 inflammasome (Chen et al., 2021). These anti-inflammatory and antioxidant effects further support GA’s potential in DR therapy and provide a new perspective for investigating its pathogenesis.

Cataracts are similarly associated with diabetes, and their pathogenesis involves mutations and physicochemical damage to lens proteins, which reduce protein stability and promote cataract formation (Islam et al., 2022). Incidence increases with age (Vision, 2020, 2000), and surgery remains the only definitive treatment. Due to limited access to surgical resources, pharmacological interventions have become a potential alternative (Lee and Afshari, 2023). Recent studies have explored combination therapies using anti-inflammatory and hypoglycemic agents to prevent or delay diabetes-related eye diseases, including cataracts and DR. In such strategies, GA often serves as a functional carrier to enhance the delivery efficiency and stability of compounds like curcumin, while its own anti-inflammatory and antioxidant properties contribute synergistically to therapeutic effects (Ganugula et al., 2023). Islam, S. et al. further investigated the anti-cataract potential of GA by evaluating its ability to inhibit γ-lens protein aggregation. They found that GA, in combination with clonal drugs, effectively prevented heat-induced protein denaturation and aggregation by binding to hydrophobic sites on γ-lens proteins. Molecular docking revealed two primary binding sites—the NC pocket and the NC tail region—which overlap with the binding site of the protective mini-α-crystallin chaperone MAC peptide, thus mimicking its function. Additionally, GA’s antioxidant properties may help delay cataract progression (Islam et al., 2022). GA demonstrates therapeutic value in ocular chronic diseases such as diabetic retinopathy and cataracts by inhibiting angiogenesis, oxidative stress, and abnormal protein aggregation (Table 1).

#### 4.3.2 GA and polycystic kidney disease

Autosomal dominant polycystic kidney disease (ADPKD) is a common inherited kidney disorder (Bergmann et al., 2018), ranking as the fourth most prevalent single-gene inherited kidney disease leading to end-stage renal disease (ESRD) worldwide (Chapman et al., 2015). ADPKD is characterized by the formation of cysts due to abnormal proliferation of renal tubular epithelial cells and fluid accumulation, resulting in progressive decline in renal function and eventual ESRD (Chebib et al., 2018). Ganguly, R. et al. investigated the effects of GA in an *in vitro* PKD cyst growth model. Using MTT assays, they confirmed that GA was non-cytotoxic to MDCK cells at concentrations of 0.5–2.5 μM, whereas 5 μM inhibited cell viability. GA significantly suppressed proliferation and cyst expansion in both MDCK and Pkd1 mutant cells, primarily by inhibiting ERK1/2 and mTOR/S6K phosphorylation, while at 2.5 μM it activated energy regulatory pathways by upregulating AMPK phosphorylation. This study thus confirmed both the antiproliferative effects of GA and its role in AMPK signaling (Khunpatee et al., 2022). Building on these findings, Zhang, J. et al. validated GA’s effects on ADPKD using both *in vitro* cellular models and animal experiments. They demonstrated that GA interfered with cell cycle progression and protein synthesis, thereby slowing aberrant proliferation of cystic epithelial cells via modulation of the ERK/mTOR/S6K pathway. Moreover, GA enhanced cellular energy homeostasis through the AMPK pathway, further inhibiting cyst formation (Zhang J. et al., 2023). These results collectively suggest that Garcinia Cambogia may serve as a promising plant-derived therapeutic candidate for ADPKD, though further clinical trials are needed to confirm its efficacy.

#### 4.3.3 GA and systemic lupus erythematosus

Systemic lupus erythematosus (SLE) is a chronic inflammatory disease characterized by loss of central and peripheral immune tolerance, production of autoantibodies, accumulation of immune complexes, and extensive tissue damage due to leukocyte infiltration (Mohan and Putterman, 2015; Moulton and Tsokos, 2015; Tsokos et al., 2016). Current SLE management relies on long-term administration of NSAIDs, corticosteroids, antimalarials, and cytotoxic agents. However, these treatments face challenges such as drug resistance and adverse effects associated with prolonged use of immunosuppressants (Murphy and Isenberg, 2019). Ganguly, R. et al. explored a novel lymphatic-targeted cyclosporine nanoparticle, specifically a biodegradable, CD71-targeting nanoparticle (P2Ns-GA), designed to enhance SLE therapy by targeting transferrin receptor 1 (CD71). GA was chemically coupled to the nanoparticle surface to facilitate targeting. Compared to unmodified nanoparticles (P2Ns), GA-modified nanoparticles (P2Ns-GA) exhibited higher binding affinity to CD3^+^ T cells and CD20^+^ B cells, significantly improving lymphatic delivery of cyclosporine while reducing nephrotoxicity. This strategy offers a promising new approach for improving therapeutic efficacy in SLE (Ganugula et al., 2020).

#### 4.3.4 GA and renal fibrosis

Renal fibrosis (RF) is a central pathological process driving the progression of chronic kidney disease (CKD) to end-stage kidney disease (ESKD), and currently, effective therapeutic strategies are limited (Liu, 2011; Yuan et al., 2022). EZH2, a histone-lysine N-methyltransferase, catalyzes the trimethylation of lysine 27 on histone H3 (H3K27me3), leading to transcriptional gene silencing (Wagener et al., 2010). Targeting EZH2 has been shown to regulate renal epithelial-mesenchymal transition (EMT) and thereby slow the progression of renal fibrosis (Zhou et al., 2018). Tao, S. et al. investigated the mechanism by which GA mitigates renal fibrosis through epigenetic regulation of EZH2. They found that GA promotes Smad7 transcription by reducing EZH2 and H3K27me3 levels, which in turn suppresses the TGF-β/Smad3 signaling pathway, attenuating renal fibrosis. Moreover, GA dose-dependently alleviated UUO- and FA-induced renal injury and fibrosis. GA also inhibited EMT in renal fibrosis, as evidenced by decreased α-SMA and increased E-cadherin expression. These findings suggest that GA may serve as a potential therapeutic agent for preventing or treating renal fibrosis (Tao et al., 2022).

#### 4.3.5 GA and hypertrophic scarring

Hypertrophic scar (HS) is a common fibrotic skin disorder that typically arises after abnormal wound healing (English and Shenefelt, 1999). Current treatment options are limited and often ineffective with minimal side effects. Studies have shown that GA can significantly reduce scar formation. The mechanism involves downregulation of vascular endothelial growth factor receptor 2 (VEGFR2), fibroblast growth factor receptor 1 (FGFR1) and their phosphorylated forms (p-VEGFR2, p-FGFR1), as well as transforming growth factor-beta 1 (TGF-β1) and connective tissue growth factor (CTGF). By modulating these targets, GA reduces collagen deposition, neovascularization, and inflammatory cell infiltration in scar tissue, highlighting its potential as a therapeutic candidate for preventing and treating hypertrophic scars (Jun et al., 2021).

In the realm of renal and fibrotic diseases, GA intervenes in cyst growth in PKD, reverses renal fibrosis, and precisely modulates immune responses in lupus (Tables 2, 3). Its intervention strategies and common mechanisms across chronic diseases are depicted in Figure 4.

**TABLE 2:** Pharmacological effects of GA in renal chronic diseases.

Disease	Core mechanism	Observed therapeutic effect	Refs
Autosomal Dominant Polycystic Kidney Disease (ADPKD)	(1) Inhibits ERK1/2 and mTOR/S6K phosphorylation, blocking abnormal proliferation of cystic epithelial cells (2) Upregulates AMPK phosphorylation, improving cellular energy homeostasis	Delays renal cyst enlargement, retards the deterioration of renal function	Khunpatee et al. (2022), Zhang et al. (2023b)
Renal Fibrosis (RF)	(1) Reduces EZH2 and H3K27me3 levels, promoting Smad7 transcription, thereby suppressing the TGF-β/Smad3 signaling pathway (2) Regulates the EMT process (↑E-cadherin, ↓α-SMA)	Alleviates renal fibrosis, reverses renal tubular epithelial-mesenchymal transition, protects renal structure	Tao et al. (2022)

This table summarizes the therapeutic mechanisms and effects of Gambogic Acid (GA) in chronic renal diseases, focusing on its role in inhibiting abnormal cell proliferation and fibrosis. Abbreviations: ADPKD, autosomal dominant polycystic kidney disease; RF, renal fibrosis; EMT, epithelial-mesenchymal transition.

**TABLE 3:** Pharmacology & applications of GA: skin and fibrotic diseases.

Disease	Core mechanism/application strategy	Observed therapeutic effect/potential advantage	Refs
Systemic Lupus Erythematosus (SLE)	Application strategy: GA-conjugated nanoparticles (P2Ns-GA) specifically target CD71^+^ lymphocytes (CD3^+^ T cells and CD20^+^ B cells)	Enhances lymphatic delivery of cyclosporine, suppresses autoimmune response, while reducing nephrotoxicity at the source	Ganugula et al. (2020)
Hypertrophic Scarring (HS)	Core mechanism: Downregulates the expression of VEGFR2, FGFR1, TGF-β1, and CTGF	Reduces collagen deposition, neovascularization, and inflammatory cell infiltration, effectively inhibiting scar formation	Jun et al. (2021)

This table outlines both the direct molecular mechanisms and innovative application strategies of Gambogic Acid (GA) in treating skin fibrotic and autoimmune diseases. Abbreviations: SLE, systemic lupus erythematosus; HS, hypertrophic scarring; VEGFR2, vascular endothelial growth factor receptor 2; FGFR1, fibroblast growth factor receptor 1; TGF-β1, transforming growth factor-beta 1; CTGF, connective tissue growth factor.

Across these diverse chronic diseases, a common theme emerges: GA exerts its therapeutic effects by mitigating the sustained cellular damage and aberrant signaling driven by inflammation and oxidative stress. However, the implications of this capability reach their zenith in the context of cancer. It is now unequivocally established that the very same processes—chronic inflammation serving as a tumor promoter and oxidative stress causing genomic instability—constitute the fundamental “soil” that nurtures the initiation, progression, and metastasis of malignancies. Thus, the stage is now set to examine GA’s most profound application: its multi-targeted war on cancer, where the mechanisms explored here are not merely protective but are weaponized to dismantle the tumor ecosystem itself.

### 4.4 Anti-cancer effects

The preceding sections have meticulously charted the pharmacological journey of Gambogic Acid (GA): from its frontline role in directly eliminating pathogens (Chaiyakunvat et al., 2016; Li et al., 2022b) and calming the hyperinflammatory host response (Ding et al., 2021; Fu et al., 2022), to its strategic intervention in the smoldering landscape of chronic diseases—such as diabetic retinopathy (Cui et al., 2018) and polycystic kidney disease (Khunpatee et al., 2022)—by quenching persistent oxidative stress and inflammation. This narrative now converges on its most profound implication: the multi-faceted war against cancer. GA’s anti-cancer prowess is not a discrete function but the culmination of its integrative biology. The very mechanisms that underpin its efficacy—particularly its mastery over the NF-κB/Nrf2 axis (Chen et al., 2021; Ding et al., 2021) and its capacity to induce metabolic and oxidative stress in pathological cells—are here amplified and repurposed to orchestrate a coordinated assault. This assault ranges from epigenetic reprogramming via non-coding RNAs (Li Y. et al., 2022; Lin et al., 2020; Wang M. et al., 2023) and the activation of a multi-modal cell death network (Joha et al., 2023; Wang S. et al., 2023; Zhang D. et al., 2024) to the systemic remodeling of the tumor microenvironment (Hatami et al., 2020; Huang et al., 2023; Xu et al., 2022)and the reversal of drug resistance (Mei et al., 2024; Wang et al., 2022a; Yu et al., 2022). In essence, cancer represents the arena where GA’s multi-target nature is fully deployed (Wang and Chen, 2012), transforming it from a broad-spectrum agent into a precise orchestrator of tumor suppression. For a systematic overview of the molecular targets and functional outcomes of these multifaceted mechanisms, readers are referred to Tables 2, 3.

The journey of GA as a multi-targeted anticancer agent begins by confronting the very foundations of cancer: the intertwined pathways of chronic inflammation and oxidative stress. As foreshadowed in our narrative of its anti-inflammatory actions, GA directly counteracts the pro-tumorigenic milieu by inhibiting key pathways such as NF-κB and STAT3, thereby suppressing the release of pro-inflammatory factors (e.g., IL-6, TNF-α) that drive cell proliferation, angiogenesis, and evasion of apoptosis. Concurrently, its established antioxidant capacity allows it to disrupt the “oxidative vicious cycle,” mitigating the DNA damage, genomic instability, and oncogenic mutations (e.g., p53 inactivation) caused by excessive ROS. This foundational disruption of the cancer-promoting environment enables GA’s broader, multi-targeted attack, which we will now deconstruct across epigenetic, cell death, and tumor microenvironment axes.

#### 4.4.1 The central role of epigenetic regulation in GA against cancer

As a natural small-molecule compound, GA has demonstrated multidimensional anticancer effects across various malignant tumors by targeting non-coding RNA networks and epigenetic regulatory systems. Recent studies reveal that its core mechanism of action operates at the epigenetic level, forming a multilevel intervention network through precise regulation of miRNA, circRNA, lncRNA, and other molecules, providing a critical theoretical basis for the development of novel anticancer therapeutics.

##### 4.4.1.1 Cell cycle and proliferation regulation

In a gastric cancer model, GA downregulated circ_ASAP2, thereby reducing its sequestration of miR-33a-5p, which led to a significant inhibition of CDK7 kinase activity (Lin et al., 2020). This dual regulation induced cell cycle arrest at the G1/S checkpoint and substantially decreased tumor cell proliferation. In colorectal cancer, GA activated the miR-199a-3p-mediated inhibition of the Wnt/β-catenin pathway, promoting efficient β-catenin ubiquitination and degradation, thereby effectively suppressing the self-renewal capacity of the CD133+/CD44+ cancer stem cell subpopulation (Li Y. et al., 2022).

##### 4.4.1.2 Molecular blockade of metastatic invasion

Regarding metastatic bladder cancer, GA directly targeted the key EMT regulator ZEB1 by upregulating miR-205-5p, which decreased ZEB1 protein expression, restored the E-cadherin/N-cadherin ratio, blocked the EMT pathway, and reduced cancer cell invasion and migration. This intervention also enhanced the sensitivity of tumor cells to cisplatin (Mei et al., 2024). In melanoma models, GA exerted antitumor effects by upregulating lncRNA MEG3. Knockdown of MEG3 enhanced melanoma metastasis, whereas GA restored MEG3 activity, significantly inhibiting tumor cell invasion and metastasis (Wang M. et al., 2023).

##### 4.4.1.3 Regulation of autophagy-apoptosis dynamic balance

Studies in melanoma have confirmed that ferroptosis and autophagy act synergistically in GA-induced melanoma cell death. GA induces autophagy by downregulating lncRNA NEAT1, activating AMPK, and indirectly inhibiting the phosphorylation of downstream mTOR proteins. Concurrently, the downregulation of lncRNA NEAT1 impairs the direct binding of SLC7A11 to GPX4, leading to decreased intracellular cystine levels and reduced glutathione synthesis. This, in turn, inhibits GPX4 activity and triggers ferroptotic cell death (Wang et al., 2022b). In a gastric cancer model, GA exerted antitumor effects by upregulating miR-26a-5p and downregulating Wnt5a. The coordinated regulation of miR-26a-5p and Wnt5a contributed to the inhibition of gastric cancer cell growth and the promotion of apoptosis. Specifically, miR-26a-5p negatively regulated Wnt5a expression by directly binding to its 3′-UTR region, highlighting a key epigenetic mechanism in the anticancer effect of GA (Zhang Z. et al., 2021).

GA achieves precise modulation of the cell cycle, EMT, autophagy–apoptosis balance, and tumor microenvironment by constructing a circRNA–miRNA–lncRNA interaction network with its key epigenetic mechanisms summarized in Table 4. Its mechanism demonstrates both cancer-specific and pathway-crossing features, deepening our understanding of the epigenetic regulation by natural products and providing theoretical support for novel anticancer strategies targeting “epigenetic hubs.” Future research should aim to construct a dynamic regulatory map of the GA epigenetic network and optimize its clinical translation pathway.

**TABLE 4:** Epigenetic regulatory mechanisms of GA in cancer.

Biological process	Core mechanism/targets	Key outcomes	Cancer models (Examples)	Refs
Cell cycle and proliferation	↓ circ_ASAP2 → ↑ miR-33a-5p → ↓ CDK7 kinase activity	G1/S phase arrest, inhibited proliferation	Gastric Cancer (GC)	Lin et al. (2020)
	↑ miR-199a-3p → ↓ Wnt/β-catenin pathway → ↑ β-catenin ubiquitination	Inhibited self-renewal of CD133+/CD44+ cancer stem cells	Colorectal Cancer (CRC)	Li et al. (2022c)
Metastasis and invasion (EMT)	↑ miR-205-5p → ↓ ZEB1 → ↑ E-cadherin/↓ N-cadherin	Blocked EMT, reduced invasion and migration, enhanced cisplatin sensitivity	Bladder Cancer (BCa)	Mei et al. (2024)
	↑ lncRNA MEG3	Inhibited tumor cell invasion and metastasis	Melanoma (MEL)	Wang et al. (2023a)
Autophagy and cell death	↓ lncRNA NEAT1 → Activates AMPK → ↓ mTOR phosphorylation → Induces autophagy	Synergistic induction of autophagic and ferroptotic cell death	Melanoma (MEL)	Wang et al. (2022b)
	↓ lncRNA NEAT1 → Impairs SLC7A11/GPX4 binding → ↓ glutathione synthesis → ↓ GPX4 activity → Triggers ferroptosis			
	↑ miR-26a-5p → Binds Wnt5a 3′-UTR → ↓ Wnt5a expression	Inhibition of cancer cell growth and promotion of apoptosis	Gastric Cancer (GC)	Zhang et al. (2021a)

This table outlines the epigenetic regulatory mechanisms by which Gambogic Acid (GA) exerts its anticancer effects, primarily through non-coding RNAs. These findings highlight GA’s potential as a multi-target epigenetic regulator, offering novel strategies for cancer therapy targeting non-coding RNAs. Abbreviations: GC, gastric cancer; CRC, colorectal cancer; BCa, bladder cancer; MEL, melanoma; EMT, epithelial-mesenchymal transition.

#### 4.4.2 Non-epigenetic regulation in GA anticancer

##### 4.4.2.1 Mechanism of GA-induced cancer cell death

**FIGURE 5:**
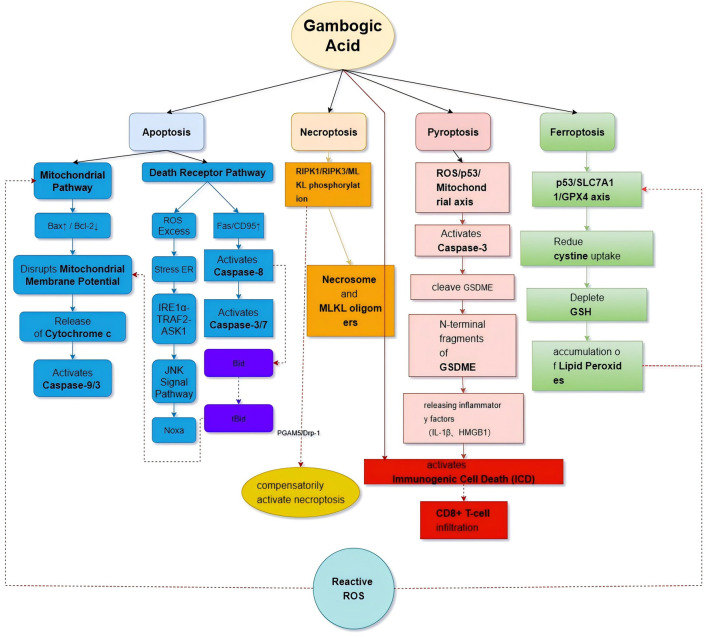
Network of multiple cell death mechanisms induced by GA.

Recent studies have demonstrated that GA exhibits significant antitumor effects across various malignancies by regulating distinct apoptotic pathways. GA induces a multidimensional network of programmed cell death, including apoptosis, necroptosis, pyroptosis, and ferroptosis. These pathways exhibit significant crosstalk, collectively constituting its potent tumor-killing efficacy (Table 5; Figure 5).

**TABLE 5:** Mechanisms of GA-induced programmed cell death.

Cell death mode	Core signaling pathway/mechanisms	Key molecular events	Cancer models (Examples)	Refs
Apoptosis	Mitochondrial pathway: ↑ Bax/↓ Bcl-2 → ΔΨm loss → Cytochrome c release → Caspase-9/3 activation	Irreversible apoptosis	GC, HCC	Joha et al. (2023), Liu et al. (2023b), Wang et al. (2022a)
​	Death receptor pathway: ↓ Microtubule polymerization → Activates NF-κB → ↑ Fas (CD95) → Caspase-3/7 activation	Apoptosis induction	MDS	Zhong et al. (2024)
​	ER Stress pathway: ↑ ROS → ER stress → IRE1α-TRAF2-ASK1 complex → ↑ Noxa → Noxa-dependent apoptosis	Apoptosis induction	OSCC, PCA	Cheng et al. (2024), Wu et al. (2023)
Necroptosis	Activates RIPK1/RIPK3/MLKL pathway → Phosphorylation of RIPK1, RIPK3, MLKL → Necrosome formation	Cross-regulation with mitochondrial apoptosis	GC	Wang et al. (2023b)
Pyroptosis	ROS/p53/Mitochondrial axis → Caspase-3 activation → Cleaves GSDME → Plasma membrane pores; Releases IL-1β, HMGB1	Immunogenic Cell Death (ICD), activates anti-tumor immunity	OC, CRC	Xu et al. (2022), Zhang et al. (2024b)
Ferroptosis	p53/SLC7A11/GPX4 axis → ↓ Cystine uptake → ↓ GSH and ↑ Lipid peroxidation → GPX4 inhibition	Synergistic with mitochondrial apoptosis	OS, MEL	Liu et al. (2023c), Wang et al. (2022b)

This table summarizes the diverse programmed cell death pathways induced by Gambogic Acid (GA) in cancer cells. The ability of GA, to activate multiple cell death modes simultaneously highlights its potential to overcome drug resistance by bypassing single-pathway dependencies. Abbreviations: MDS, myelodysplastic syndrome; OSCC, oral squamous cell carcinoma; PCA, prostate cancer; OC, ovarian cancer; OS, osteosarcoma; GSDME, gasdermin E; ICD, immunogenic cell death.

In the mitochondrial apoptosis pathway, GA dynamically regulates the balance of Bcl-2 family proteins. Specifically, GA upregulates the pro-apoptotic protein Bax while inhibiting the anti-apoptotic protein Bcl-2, leading to alterations in mitochondrial membrane potential and the release of cytochrome C, which activates the Caspase-9/3 cascade. This mechanism has been validated in gastric cancer (Joha et al., 2023), hepatocellular carcinoma (Liu S. et al., 2023; Wang et al., 2022a), and other tumors. In HCC models, GA not only significantly elevated the Bax/Bcl-2 ratio but also amplified apoptotic signaling via ROS-mediated oxidative damage (Liu S. et al., 2023).

The death receptor pathway exhibits tissue-specific features. In a myelodysplastic syndrome (MDS) model, GA triggers apoptosis by activating the Fas/CD95 pathway (Zhong et al., 2024). Specifically, it inhibits microtubule polymerization, activates the NF-κB signaling pathway, and upregulates Fas (CD95) expression, resulting in enhanced caspase-3/7 activity. Experiments revealed that soluble microtubule proteins increased while polymerized microtubules decreased in GA-treated MDS cells, with enhanced NF-κB binding to microtubules further promoting apoptotic signaling. In oral cancer (Cheng et al., 2024) and prostate cancer (Wu et al., 2023), GA acts through the ROS/ER stress–JNK signaling axis. In oral squamous cell carcinoma (OSCC), GA-induced ROS triggers ER stress, activating the JNK pathway and upregulating the pro-apoptotic protein Noxa via the IRE1α–TRAF2–ASK1 complex, ultimately inducing Noxa-dependent apoptosis (Cheng et al., 2024).

Notably, in esophageal squamous cell carcinoma (ESCC), GA activates the PTEN/PI3K/AKT/mTOR pathway through the mitochondria-dependent apoptosis pathway, effectively inhibiting cancer cell proliferation while promoting apoptosis (Yu et al., 2020). This multi-target, multi-pathway feature underscores GA’s potential as a broad-spectrum antitumor agent and highlights the specificity of its signal transduction in different tumor microenvironments, providing a theoretical basis for precision therapy.

Beyond classical apoptosis, GA also activates novel programmed cell death modes such as necroptosis, pyroptosis, and ferroptosis, forming a multidimensional antitumor network. These mechanisms interact with classical apoptotic pathways to enhance GA’s overall tumor-killing efficiency.

In gastric cancer models, GA induces necroptotic vesicle formation by activating the phosphorylation of key components in the RIPK1/RIPK3/MLKL pathway, which is regulated via crosstalk between necroptosis and apoptosis. Levels of RIPK1, RIPK3, and MLKL phosphorylation significantly increased after GA treatment, a process dependent on mitochondrial fission proteins PGAM5 and Drp-1 (Wang S. et al., 2023). Importantly, there is a dynamic balance between necroptosis and mitochondrial apoptosis: when the mitochondrial pathway is blocked, GA compensates by activating necroptosis through enhanced RIPK1 signaling, reinforcing the dual-death pathway strategy to maximize tumor cell eradication.

GA also amplifies the cascade between pyroptosis and mitochondrial apoptosis. Activation of caspase-3 via the ROS/p53/mitochondrial signaling axis cleaves the execution protein GSDME, forming plasma membrane pores that trigger pyroptosis in ovarian cancer cells (Zhang D. et al., 2024). This process synergizes with the classical mitochondrial apoptotic pathway: caspase-9 activation via the mitochondrial pathway enhances GSDME cleavage by caspase-3. Furthermore, inflammatory mediators (e.g., IL-1β, HMGB1) and tumor antigens released during pyroptosis stimulate immunogenic cell death, promoting CD8^+^ T cell infiltration and dendritic cell maturation. In colorectal cancer models, GA increases the CD3+/CD8+ T cell ratio within the tumor microenvironment, revealing a synergistic antitumor mechanism combining pyroptosis and adaptive immune responses (Xu et al., 2022).

In osteosarcoma (Liu Z. et al., 2023) and melanoma (Wang et al., 2022b), GA exerts metabolic synergy between ferroptosis and apoptosis. It depletes glutathione (GSH) and promotes lipid peroxidation via inhibition of cystine uptake through the p53/SLC7A11/GPX4 axis. At the metabolic level, ferroptosis integrates with mitochondrial apoptosis: GA-induced ROS bursts both enhance Bax/Bcl-2 imbalance and accelerate ferroptosis by inhibiting GPX4. Experimental blockade of mitochondrial apoptosis (via Bcl-2 overexpression) and ferroptosis (via Ferrostatin-1 preconditioning) significantly reduced GA cytotoxicity, highlighting the synergistic interaction of these death pathways.

GA constructs a multidimensional programmed cell death network through spatiotemporal-specific regulation. The mitochondrial pathway (Bax/Bcl-2–Caspase9/3) and the death receptor pathway (Fas/CD95–Caspase8) serve as core apoptotic engines, cross-talking with necroptosis (RIPK1/3–MLKL), pyroptosis (Caspase3–GSDME), and ferroptosis (SLC7A11–GPX4) to establish a multilevel death network. For instance, caspase-8 from the death receptor pathway cleaves Bid to tBid, promoting cytochrome C release via the mitochondrial pathway, while lipid peroxides from ferroptosis enhance mitochondrial apoptosis through oxidative modification of Bcl-2. This multidimensional death network allows GA to overcome single-pathway drug resistance, offering a novel strategy for precision tumor therapy.

GA also modulates autophagy homeostasis in a tumor type-dependent manner, exerting bidirectional effects. In hepatocellular carcinoma (Wang et al., 2022a) and prostate cancer (Wu et al., 2023) models, GA inhibits protective autophagy to enhance chemosensitivity while inducing cytotoxic autophagy to synergistically promote tumor cell death.

In hepatocellular carcinoma, GA blocks autophagosome–lysosome fusion, leading to abnormal accumulation of intracellular autophagosomes. This is accompanied by an increased LC3-II/LC3-I ratio and upregulation of Beclin1, effectively inhibiting tumor protective autophagy. This persistent blockage enhances the chemosensitivity of HCC cells to adriamycin, providing a theoretical basis for clinical combination therapy (Wang et al., 2022a).

In prostate cancer, GA induces ROS bursts that trigger ER stress and activate the JNK signaling pathway, initiating autophagy as indicated by simultaneous accumulation of LC3-II and p62. Autophagy acts as a “double-edged sword”: short-term activation aids stress adaptation, whereas persistent autophagy exerts cytotoxic effects. Co-treatment with the autophagy inhibitor chloroquine blocks protective autophagy and increases apoptosis, confirming the dynamic regulation of autophagy in GA-mediated pro-apoptotic effects (Wu et al., 2023).

These findings reveal how GA influences tumor fate through spatiotemporal-specific autophagy regulation, providing a foundation for combined therapeutic strategies that exploit autophagy–apoptosis crosstalk. Notably, the synergistic use of autophagy inhibitors with GA presents innovative opportunities to overcome tumor drug resistance.

##### 4.4.2.2 Mechanisms of GA inhibition of tumor proliferation and metastasis

Cell cycle regulation is a fundamental mechanism governing tumorigenesis and progression, and its dysregulation is closely associated with uncontrolled proliferation in many malignant tumors. Current studies indicate that targeted blockade of specific cell cycle phases plays a crucial role in tumor therapy, with particular emphasis on the G0/G1 and G2/M phases. During the G0/G1 phase, cell cycle progression can be effectively halted by inhibiting CDK4/6 kinase activity, a mechanism that has shown significant therapeutic potential in solid tumors such as non-small cell lung cancer (NSCLC) (Shen et al., 2020) and cervical cancer (Niu et al., 2023). In NSCLC, GA induces G1-phase arrest through a dual mechanism: first, it inhibits the phosphorylation activity of the cyclin D–CDK4/6 complex, thereby blocking RB protein phosphorylation; second, it upregulates the p53/p21 signaling pathway, collectively enforcing cell cycle arrest at the G1 checkpoint (Shen et al., 2020).

At the G2/M phase, a critical regulatory point for mitosis, the Cyclin B1/CDK1 complex governs the initiation of cell division. Studies in glioblastoma models have demonstrated that GA effectively induces G2/M arrest by modulating Cyclin B1 expression and altering CDK1 activation (Dong et al., 2022). This blockade not only inhibits tumor cell mitosis but also enhances apoptosis of abnormal cells by prolonging DNA damage checkpoint activation.

These findings clarify the molecular mechanisms by which GA inhibits tumor proliferation and lay the foundation for the development of targeted therapies against cyclins and CDKs. The differential sensitivity of tumor types to specific cell cycle checkpoints highlights the need for tumor-specific therapeutic strategies, selecting the most appropriate phase for intervention. Epithelial-mesenchymal transition (EMT), a central driver of tumor invasion and metastasis, has become a critical target in anti-tumor therapy. Studies indicate that the metastatic potential of tumor cells can be effectively reversed by modulating EMT-related molecular markers and key signaling pathways, particularly the balance of E-cadherin/N-cadherin/Vimentin and the activation state of PI3K/Akt and Wnt/β-catenin pathways. At the molecular marker level, GA exhibits bidirectional regulation: in bladder cancer (Mei et al., 2024) and melanoma (Wang M. et al., 2023) models, it restores intercellular adhesion by upregulating the epithelial marker E-cadherin while significantly downregulating mesenchymal markers N-cadherin and Vimentin. This effect was especially pronounced in melanoma, where GA not only inhibited mesenchymal transformation through activation of the p53/SLC7A11/GPX4 axis but also enhanced anti-tumor activity via modulation of the iron death pathway (Wang et al., 2022b). These results reveal the multidimensional mechanism by which GA regulates EMT and underscore the heterogeneity of EMT networks across tumor types, providing a framework for precise targeting of EMT drivers in the tumor microenvironment to curb metastasis.

Inhibition of tumor cell migration and invasion is a key component of blocking metastasis and involves both extracellular matrix (ECM) remodeling and regulation of invasion-related signaling pathways. Targeting matrix metalloproteinases (MMPs) and key matrix-regulated pathways has been shown to significantly impair tumor invasiveness, particularly through inhibition of MMP-2/MMP-9 activity and activation of the RORB/EMILIN1 pathway.

At the stromal degradation level, GA suppresses tumor migration by downregulating MMPs. In malignant glioma, GA reduces MMP expression, thereby inhibiting cell migration (Dong et al., 2022). In colorectal cancer, GA decreases both gene expression and enzymatic activity of MMP-2/MMP-9, limiting type IV collagen degradation at the basement membrane (Rech et al., 2021). Similarly, in melanoma, GA upregulates E-cadherin, downregulates N-cadherin, Vimentin, MMP-2, and MMP-9, and enhances cell-matrix adhesion, forming a dual anti-invasive barrier that blocks EMT and invasion (Wang M. et al., 2023).

In terms of invasion-related signaling, GA demonstrates cross-cancer regulatory potential. In hepatocellular carcinoma, GA inhibits nuclear translocation of Smad2/3 by blocking TGF-β receptor type II (TβRII) phosphorylation, leading to downregulation of tissue inhibitors of metalloproteinases (TIMPs) and fibronectin. This precise modulation of the TGF-β pathway hinders pseudopod formation and reverses the pro-metastatic tumor microenvironment (Liu S. et al., 2023).

Collectively, these studies reveal the multidimensional mechanisms by which GA inhibits tumor motility, emphasizing its unique ability to achieve anti-metastatic effects through both ECM metabolic reprogramming and signaling pathway cross-regulation. The selective targeting of MMPs or matrix stabilizers based on tumor type provides novel strategies for developing precision anti-metastatic therapies.

#### 4.4.3 Antiangiogenic mechanisms: multi-targeted effects of GA

Recent studies indicate that the natural compound GA exerts significant anti-angiogenic activity through a multi-targeted mechanism involving two core regulatory systems:

##### 4.4.3.1 VEGF signaling pathway intervention

GA inhibits the vascular endothelial growth factor (VEGF) system via a dual mechanism. In non-small cell lung cancer models, GA specifically downregulates VEGFR2 expression, effectively suppressing tumor angiogenesis (Hatami et al., 2020). Similar VEGF-mediated anti-angiogenic effects of GA have been observed in multiple myeloma (Yu et al., 2022), malignant glioma (Dong et al., 2022), and colorectal cancer (Huang et al., 2023). Mechanistic studies in non-small cell lung cancer revealed that GA suppresses tumor angiogenesis by inhibiting the YAP/STAT3 signaling axis, thereby reducing VEGFR2 expression and effectively cutting off the tumor blood supply (Hatami et al., 2020).

##### 4.4.3.2 Hypoxic microenvironment regulation

GA also demonstrates a unique ability to modulate the hypoxic tumor microenvironment. It blocks hypoxia-induced angiogenesis by targeting hypoxia-inducible factor-1α (HIF-1α), a mechanism particularly evident in multiple myeloma (Yu et al., 2022). This property allows GA to overcome the limitations of traditional anti-angiogenic drugs that act on a single target, providing a broader spectrum of anti-tumor activity. The multi-target synergistic action of GA not only enhances its anti-angiogenic efficacy but also reduces the likelihood of drug resistance associated with single-target therapies. By acting on multiple levels—from receptor inhibition to microenvironmental regulation—GA exemplifies the advantages of a natural multi-targeted anti-angiogenic agent and offers an important theoretical foundation for the development of novel tumor treatment strategies.

Thus, beyond directly inducing cell death, GA effectively suppresses tumor proliferation and dissemination by arresting the cell cycle, reversing EMT, inhibiting MMP activity, and blocking tumor angiogenesis (Table 6).

**TABLE 6:** Mechanisms of GA in inhibiting tumor proliferation, invasion, and metastasis.

Biological process	Core mechanism/targets	Key outcomes	Cancer models (Examples)	Refs
Cell cycle arrest	↓ CDK4/6 kinase activity; ↑ p53/p21 pathway	G0/G1 phase arrest	Lung Cancer (LC)	Shen et al. (2020)
	Disrupts Cyclin B1/CDK1 complex dynamics	G2/M phase arrest	Glioma (GBM)	Dong et al. (2022)
EMT and invasion	↑ E-cadherin; ↓ N-cadherin, Vimentin	Re-established cell adhesion, reversed mesenchymal phenotype	BCa, MEL	Mei et al. (2024), Wang et al. (2023a)
ECM remodeling and migration	↓ MMP-2/MMP-9 expression and activity	Inhibited migration and invasion	GBM, CRC, MEL	Dong et al. (2022), Rech et al. (2021), Wang et al. (2023a)
	↓ TβRII phosphorylation → ↓ Smad2/3 nuclear translocation → ↓ TIMPs, Fibronectin	Hindered pseudopod formation, reversed pro-metastatic TME	HCC	Liu et al. (2023b)
Anti-angiogenesis	↓ YAP/STAT3 axis → ↓ VEGFR2 expression	Suppressed tumor angiogenesis	LC	Hatami et al. (2020)
	Targets HIF-1α → Inhibits hypoxia-induced angiogenesis	Broad-spectrum anti-angiogenic activity	Multiple Myeloma (MM)	Yu et al. (2022)

This table delineates the mechanisms by which Gambogic Acid (GA) inhibits tumor proliferation, invasion, and metastasis, focusing on cell cycle, EMT, and microenvironmental regulation. GA’s coordinated targeting of multiple hallmarks of cancer progression underscores its promise as a broad-spectrum therapeutic agent against advanced and metastatic cancers. Abbreviations: LC, lung cancer; GBM, glioma; HCC, hepatocellular carcinoma; ECM, extracellular matrix; MMP, matrix metalloproteinase; TβRII, TGF-β, receptor type II; TIMP, tissue inhibitor of metalloproteinases.

#### 4.4.4 Immunoregulatory mechanisms: multidimensional immunoregulatory networks of GA

Recent studies have revealed that GA possesses unique immunomodulatory functions, acting on both the innate and adaptive immune systems to form a multilevel immunoregulatory network:

##### 4.4.4.1 Enhanced immune initiation

GA activates the immune response through a dual mechanism. In colorectal cancer (Huang et al., 2023) and triple-negative breast cancer (Li L. et al., 2022) models, GA significantly upregulates the expression of dendritic cell (DC) maturation markers CD86 and CD80, thereby enhancing antigen presentation efficiency. This effect is closely linked to GA-induced tumor cell pyroptosis, which triggers programmed cell death and releases large amounts of tumor-specific antigens. This process creates a “vaccine-in-place” effect, stimulating cytotoxic T-lymphocyte (CTL) activity and amplifying tumor-killing responses.

##### 4.4.4.2 Effector cell regulation

GA remodels T-cell subpopulations within the tumor microenvironment. In breast cancer (Li L. et al., 2022) and colorectal cancer (Huang et al., 2023; Xu et al., 2022) models, GA treatment increased CD8^+^ T-cell infiltration several-fold and reversed T-cell exhaustion by inhibiting the PD-1/PD-L1 immune checkpoint axis. This “two-pronged” regulatory strategy transforms the tumor immune microenvironment from a cold tumor to a hot tumor, enhancing the efficacy of anti-tumor immunity.

These findings demonstrate that GA overcomes the limitations of traditional immunotherapies by modulating the complete immune cycle—from antigen presentation to immune activation and effector killing. Its unique immunoregulatory properties, when combined with previously identified anti-angiogenic effects, provide a promising foundation for the development of natural product-based tumor immuno-combination therapies.

#### 4.4.5 Tumor microenvironment remodeling: systemic regulation of heterogeneity, matrix, and immune ecology by GA

The tumor microenvironment (TME) is a complex ecosystem composed of cancer cells, immune cells, stromal cells, vascular networks, and the extracellular matrix (ECM) (110). Beyond the direct elimination of tumor cells, GA exerts anti-cancer effects through multidimensional, precision-targeted reprogramming of this ecosystem, thereby inhibiting tumor progression, reversing immune suppression, and overcoming drug resistance.

Firstly, targeting tumour heterogeneity: dismantling the synergistic alliance of cancer cells. Tumor heterogeneity is a primary cause of therapeutic failure (Roerden and Spranger, 2025). GA disrupts tumor adaptability by selectively targeting functional cancer cell subpopulations. In colorectal cancer, GA suppresses the self-renewal capacity of cancer stem cell (CSC) subpopulations, the “seed cells” responsible for recurrence, metastasis, and drug resistance. This inhibition of CSC stemness directly undermines the core driver of tumor heterogeneity, limiting sustained proliferation and regeneration (Mei et al., 2024). In bladder cancer and melanoma, GA restores the E-cadherin/N-cadherin ratio, reprogramming invasive mesenchymal-like cells into a relatively quiescent epithelial-like state (Wang et al., 2022b; Wang M. et al., 2023). This reduces metastasis potential and diminishes phenotypic plasticity associated with the EMT-MET cycle.

Secondly, modulating the stroma-tumour interaction: disrupting the supportive “soil” for cancer cells. GA reshapes the tumor-supportive microenvironment by targeting stromal components. In colorectal cancer, GA alters the polarization of tumor-associated macrophages (TAMs) (Zhang D. et al., 2021), while in liver cancer models, it suppresses cancer-associated fibroblast (CAF) activation (Zhong et al., 2024). These effects dismantle the physical and chemical scaffolds that sustain malignant tumor growth, impairing the tumor’s capacity to thrive within its niche.

Finally, multidimensional immunomodulation: converting “cold tumors” into “hot tumors.” GA-induced pyroptosis and immunogenic cell death (ICD) release abundant tumor-associated antigens and danger signals (e.g., HMGB1, ATP) (Xu et al., 2022; Zhang D. et al., 2021), which efficiently promote DC maturation (Li L. et al., 2022; Liu et al., 2021) and generate an “*in situ* vaccination” effect that initiates the immune cycle. Simultaneously, GA enhances CD8^+^ T cell infiltration within the TME (Liu et al., 2021; Liu Z. et al., 2023) and downregulates PD-1/PD-L1 checkpoint expression, reversing T-cell exhaustion and restoring the cytotoxic capacity of infiltrating immune cells (Li L. et al., 2022; Liu Z. et al., 2023).

These findings highlight GA’s systemic remodeling of the TME, integrating tumor heterogeneity suppression, stromal reprogramming, and immune activation. Future studies should employ single-cell sequencing and spatial transcriptomics to map GA’s dynamic TME modulation at higher resolution. Additionally, exploring combination strategies with modern immunotherapies, such as immune checkpoint inhibitors, may unlock more effective and clinically translatable anti-cancer strategies.

#### 4.4.6 Reversing resistance mechanisms: a multi-targeted resistance regulatory system for GA

The characteristics of the tumor microenvironment, such as cancer stem cells (CSCs), immune suppression, and the stromal barrier, are fundamental drivers of drug resistance (Liu et al., 2024). As a natural compound with broad-spectrum anticancer activity, GA demonstrates remarkable potential in reversing multidrug resistance (MDR) in cancer. Its mechanism of action involves multi-target regulation and synergistic effects, encompassing dual dimensions of drug-resistance gene modulation and chemotherapeutic sensitization, thereby forming a unique system for resistance reversal. In solid tumors, GA can reverse drug resistance by interfering with key signaling pathways and regulating the expression of drug resistance-related proteins. In hepatocellular carcinoma (HCC), GA significantly enhances the sensitivity of tumor cells to cisplatin and adriamycin by inhibiting autophagic flux (blocking autophagosome degradation), downregulating P-glycoprotein (P-gp), and suppressing the TGF-β signaling pathway (Wang et al., 2022a). In non-small cell lung cancer (NSCLC), GA improves sensitivity to cisplatin and gemcitabine by inhibiting the NF-κB and MAPK/HO-1 pathways, while reducing the expression of resistance-associated proteins such as MDR1 and RRM1 (Hatami et al., 2020; Shen et al., 2020). In breast cancer, GA overcomes endocrine therapy resistance by targeting the activity of the ERα Y537S mutant in synergy with CDK4/6 inhibitors, and by downregulating HSP90, thereby reducing tumor cell thermotolerance to photothermal therapy (Liu et al., 2021; Yang et al., 2021). In colorectal cancer (CRC), GA diminishes the stemness of CSCs through miR-199a-3p-mediated inhibition of the Wnt/β-catenin pathway (Li Y. et al., 2022), whereas in bladder cancer (BCa), GA suppresses EMT and cisplatin resistance via the miR-205-5p/ZEB1 axis, downregulating proteins such as LRP, MRP, and P-gp (Mei et al., 2024). GA also exhibits notable efficacy in hematologic malignancies. In chronic myeloid leukemia (CML), GA reverses resistance to imatinib and doxorubicin by inhibiting BCR-ABL and its downstream STAT5, ERK1/2, and Akt signaling pathways, while reducing pregnane X receptor (PXR) expression (Wang et al., 2020). In multiple myeloma (MM), GA downregulates cisplatin-resistant proteins (LRP, MRP, P-gp) by promoting ROS accumulation and activating caspase signaling. When combined with bortezomib, GA significantly enhances pro-apoptotic effects through the caspase-3/PARP pathway and inhibition of PI3K/Akt signaling (Yu et al., 2022). In summary, GA effectively reverses multidrug resistance in both solid tumors and hematologic malignancies via multiple mechanisms, including modulation of key signaling pathways (e.g., NF-κB, PI3K/Akt, Wnt/β-catenin), inhibition of drug-resistance proteins (P-gp, MDR1), epigenetic regulation (miRNAs, lncRNAs), and induction of programmed cell death (apoptosis, pyroptosis). These findings highlight GA as a powerful strategy to overcome chemoresistance in clinical settings. Collectively, the evidence confirms that GA reshapes tumor cell drug responsiveness through a triple-action mode: inhibiting drug-efflux pumps, blocking survival signals, and activating death pathways. Its multi-targeted, pathway-crossing action not only overcomes the limitations of single-pathway inhibitors but also provides a novel molecular target and theoretical framework for the design of combination therapies for drug-resistant tumors.

Notably, GA remodels the tumor microenvironment, activates anti-tumor immunity, and reverses multi-drug resistance through multi-targeted actions, providing a solid foundation for its use in combination therapies (Tables 7, 8). But it also presents its greatest clinical challenge: poor pharmacokinetics, including low aqueous solubility, non-specific tissue distribution, and rapid systemic clearance, which collectively limit the translation of its robust mechanistic potential into clinical reality. To overcome this fundamental dichotomy between potent pharmacodynamics and suboptimal pharmacokinetics, the scientific community has turned to a strategic solution: nanodelivery systems. The following section will elucidate how nanotechnology is being harnessed to tame GA’s promiscuity for precise clinical application.

**TABLE 7:** Multitargeted mechanisms and resistance reversal of GA in hematologic malignancies.

Hematologic malignancy and key targets	Core mechanisms of action	Refs
Multiple Myeloma (MM)	(1) Synergizes with bortezomib to enhance apoptosis via caspase-3/PARP pathway and PI3K/Akt inhibition. (2) Inhibits HIF-1α/VEGF pathway and downregulates oncogenic miR-21 under hypoxia. (3) Blocks CXCR4 signaling, reducing osteoclastogenesis. (4) Downregulates SIRT1 via ROS accumulation, promoting apoptosis	Pandey et al. (2014), Yang et al. (2012), Yu et al. (2022)
Chronic Myeloid Leukemia (CML)	(1) Inhibits BCR-ABL and its downstream pathways (STAT5, ERK1/2, Akt). (2) Reduces Pregnane X receptor (PXR) expression. (3) Induces proteasome inhibition, leading to caspase-3 activation and PARP cleavage	Shi et al. (2014), Wang et al. (2020)
Diffuse Large B-cell Lymphoma (DLBCL)	Induces proteasome inhibition, triggering apoptosis	Shi et al. (2015)
Acute T-cell Leukemia (T-ALL)	Induces autophagy via inhibition of the β-catenin signaling pathway	Wang et al. (2020)
Acute Myeloid Leukemia (AML)	Induces cell differentiation by upregulating p21 protein expression	Chen et al. (2014)

This table summarizes the key molecular mechanisms of GA, in hematologic malignancies. Notably, many of these mechanisms (e.g., inhibition of BCR-ABL, proteasome, P-gp) underpin its demonstrated efficacy in reversing chemoresistance in preclinical models. Abbreviations: MM, multiple myeloma; CML, chronic myeloid leukemia; DLBCL, diffuse large B-cell lymphoma; T-ALL, acute T-cell leukemia; AML, acute myeloid leukemia; PARP, poly (ADP-ribose) polymerase; PXR, pregnane X receptor.

**TABLE 8:** Immunoregulatory and resistance-reversal mechanisms of GA.

Biological Process	Core mechanism/targets	Key outcomes	Cancer models (Examples)	Refs
Immunoregulation	Immune priming: Induces pyroptosis/ICD → ↑ DC maturation markers (CD86, CD80) → Enhanced antigen presentation	“*In situ* vaccination” effect, activates CTLs	CRC, BC	Huang et al. (2023), Li et al. (2022d)
	Effector cell regulation: ↑ CD8^+^ T-cell infiltration; ↓ PD-1/PD-L1 axis	Reverses T-cell exhaustion, transforms “cold” to “hot” tumors	CRC, BC	Huang et al. (2023), Li et al. (2022d), Xu et al. (2022)
Reversing resistance	↓ Autophagic flux; ↓ P-gp; ↓ TGF-β signaling	Re-sensitization to cisplatin and adriamycin	HCC	Wang et al. (2022a)
	↑ ROS → Caspase-3/PARP activation; ↓ PI3K/Akt signaling (with bortezomib)	Enhanced pro-apoptotic effect	MM	Yu et al. (2022)
	↓ NF-κB and MAPK/HO-1 pathways; ↓ MDR1, RRM1	Enhanced sensitivity to gemcitabine and cisplatin	LC	Hatami et al. (2020), Shen et al. (2020)
	↓ ERα Y537S mutant activity (with CDK4/6 inhibitors); ↓ HSP90	Overcame endocrine therapy resistance; reduced thermotolerance	BC	Liu et al. (2021), Yang et al. (2021)
	miR-205-5p/ZEB1 axis → ↓ LRP, MRP, P-gp	Overcame cisplatin resistance	BCa	Mei et al. (2024)
	↓ BCR-ABL → ↓ STAT5, ERK1/2, Akt; ↓ PXR expression	Reversal of imatinib and doxorubicin resistance	CML	Wang et al. (2020)

This table summarizes the immunomodulatory functions of Gambogic Acid (GA) and its key mechanisms in reversing chemoresistance across various cancers. Abbreviations: DC, dendritic cell; CTL, cytotoxic T-lymphocyte; P-gp, P-glycoprotein; MDR1, multidrug resistance protein 1; LRP, lung resistance-related protein; MRP, multidrug resistance-associated protein.

#### 4.4.7 Nanosynthesis strategies: a precision revolution in GA delivery systems

GA, as a natural anti-tumor agent, faces inherent challenges including low bioavailability and limited tumor-targeting efficiency. These limitations are common among natural products with potent pharmacological activity but suboptimal physicochemical properties. Integration with nanodelivery systems has emerged as a pivotal strategy to overcome these constraints, enabling GA to achieve synergistic and enhanced anti-cancer effects across multiple tumor types. To overcome the inherent pharmacokinetic defects of GA, researchers have developed various advanced nanodelivery systems. These can be broadly categorized into those designed to enhance tumor targeting and controlled release (Table 9), and those focusing on immune activation and combination therapy (Table 10).

**TABLE 9:** GA nanodelivery systems for enhanced targeting and control.

Nanoparticle platform/strategy	Core function and mechanism	Key application and outcome	Refs
Nanoparticles (GA-NPs)	Enhances renal uptake and retention; reduces oxidative stress and improves renal function	Acute Kidney Injury (AKI): Marked renoprotective effects with excellent biosafety	Li et al. (2023)
Lactoferrin-modified liposomes (LF-lipo)	Active targeting via LRP-1 receptor; induces immunogenic cell death (ICD)	Colorectal Cancer (CRC): Inhibits liver metastasis and reduces postoperative recurrence	Wang et al. (2023c)
PLGA-CMB microbubble complex + ultrasound	Ultrasound-targeted microbubble destruction (UTMD) enhances blood-brain barrier penetration	Glioma (GBM): Increases drug concentration in tumors, induces apoptosis in glioblastoma stem cells	Dong et al. (2022)
Low-frequency ultrasound + chemical enhancers	Significantly increases transdermal permeability of GA.	Melanoma (MEL): Achieves localized high efficacy for cutaneous melanoma	Zhang et al. (2021b)
Thermosensitive hydrogel (GA-MIC-GEL)	Localized delivery and slow release at the tumor site; remodels the immune microenvironment	Oral Squamous Cell Carcinoma (OSCC): Local tumor cell killing and inhibition of distant metastasis via immune activation	Chen et al. (2022)
iRGD-modified thermosensitive hydrogel	Enhanced tumor penetration via tumor-penetrating peptide iRGD and EPR effect	Gastric Cancer (GC): Enhances tumor accumulation and suppresses metastasis	Zhang et al. (2020)

This table highlights nanodelivery systems designed to enhance the targeting and controlled release of Gambogic Acid (GA), thereby improving its pharmacokinetic profile. Abbreviations: AKI, acute kidney injury; CRC, colorectal cancer; GBM, glioma; MEL, melanoma; OSCC, oral squamous cell carcinoma; GC, gastric cancer; LRP-1, low-density lipoprotein receptor-related protein 1; UTMD, ultrasound-targeted microbubble destruction; EPR, enhanced permeability and retention effect.

**TABLE 10:** GA nanodelivery systems for immune activation and combination therapy.

Nanoparticle platform/strategy	Core function and mechanism	Key application and outcome	Refs
Cancer cell membrane-coated Nanoparticles	Mimics tumor antigens; activates dendritic cells (DCs) and enhances antigen presentation	Broad-spectrum Cancer Vaccination: Potentiates antitumor immunity	Huang et al. (2023)
G-G@HTA Self-assembled nanoparticles	Co-delivers GA and Gemcitabine (Gem) via *in vivo* self-assembly; overcomes multidrug resistance	Non-Small Cell Lung Cancer (NSCLC): Synergistic inhibition of tumor growth with reduced toxicity	Hatami et al. (2022)
Photosensitive micelles (GA@PEG-TK-ICG)	Near-infrared (NIR) light-triggered drug release; synergistic photothermal-chemotherapy	Breast Cancer (BC): Inhibits metastasis and induces mitochondrial apoptosis	Yang et al. (2021)
CD71-targeting Nanoparticles (P2Ns-GA)	Precisely targets CD71 (Transferrin Receptor) on lymphocytes (CD3^+^ T, CD20^+^ B cells)	Systemic Lupus Erythematosus (SLE): Enhances drug delivery to immune cells, reduces nephrotoxicity	Ganugula et al. (2020)
Core-shell nanocarriers (GHC NPs)	Combines chemotherapy, anti-angiogenesis and immunotherapy for triple synergy	Hepatocellular Carcinoma (HCC): Promotes DC maturation and T cell infiltration	Shao et al. (2020)
MILAN nanocomposite (Mild photothermal-immunotherapy)	GA induces ICD; Hyaluronic acid-CpG activates DCs and recruits CD8^+^ T cells	Breast Cancer (BC): Effective tumor ablation with enhanced long-term immune memory	Li et al. (2022d)

This table focuses on advanced nanodelivery platforms that leverage Gambogic Acid (GA) for immune activation and synergistic combination therapy. Abbreviations: NSCLC, non-small cell lung cancer; BC, breast cancer; SLE, systemic lupus erythematosus; HCC, hepatocellular carcinoma; ICD, immunogenic cell death; NIR, near-infrared.

The rapid development of nanomedicine platforms for other natural products—such as isorhamnetin, curcumin, and resveratrol—has provided valuable insights and a clear roadmap for advancing GA-based therapies. For example, isorhamnetin, a flavonoid with strong antioxidant and anticancer properties, suffers from poor water solubility and rapid metabolism, limiting its clinical utility. Formulation into PLGA nanoparticles or liposomes significantly improves its oral bioavailability by protecting the compound from gastrointestinal degradation and hepatic first-pass metabolism. Importantly, surface modifications, such as conjugation with RGD peptides to target tumor neovasculature, enable efficient tumor-site accumulation. This strategy not only amplifies tumor penetration and retention via the enhanced permeability and retention (EPR) effect but also reduces systemic toxicity (Rana et al., 2025).

Currently, GA-related nanodelivery systems are also being extensively researched. oral squamous cell carcinoma (OSCC), GA-loaded thermosensitive hydrogels (GA-MIC-GEL) achieve localized tumor cell killing while simultaneously activating systemic anti-tumor immunity, thereby inhibiting distant metastasis. Notably, the therapeutic effect relies predominantly on immune modulation rather than direct cytotoxicity (Chen et al., 2022). In gastric cancer (GC), thermosensitive hydrogel-loaded GA nanoparticles combined with the tumor-penetrating peptide iRGD significantly enhance tumor accumulation through the EPR effect, while concurrently suppressing metastasis and reversing chemotherapy resistance (Zhang et al., 2020). For hepatocellular carcinoma (HCC), PLGA-based nanocarriers combined with ultrasound-targeted microbubble disruption (UTMD) overcome physiological barriers, markedly increase GA delivery to tumor sites, and restore cisplatin sensitivity by inhibiting the TGF-β and NF-κB signaling pathways (Shao et al., 2020). For colorectal cancer (CRC), lactoferrin-modified liposomes (LF-lipo) enhanced tumor-selective delivery of GA by targeting low-density lipoprotein receptor-related protein 1 (LRP-1), while simultaneously inducing immunogenic cell death (ICD) and reprogramming tumor-associated macrophage (TAM) polarization, thereby activating systemic anti-tumor immunity (Wang R. et al., 2023). In non-small cell lung cancer (NSCLC), G-G@HTA nanoparticles co-delivered GA and gemcitabine (Gem) via *in vivo* self-assembly, effectively inhibiting tumor growth, overcoming multidrug resistance, and reducing systemic toxicity (Hatami et al., 2022). In breast cancer (BC), pH-responsive micelles and photosensitized nanocarriers (e.g., GA@PEG-TK-ICG) enabled near-infrared (NIR)-triggered drug release, suppressed HSP90 expression to enhance photothermal-chemotherapeutic synergy, and selectively targeted mitochondria to induce apoptosis (Yang et al., 2021). For melanoma (MEL), low-frequency ultrasound combined with chemo-enhancers significantly increased GA transdermal permeability, achieving localized high efficacy by inhibiting epithelial-mesenchymal transition (EMT) and inducing ferroptosis (iron-dependent cell death) (Zhang D. et al., 2021). Additionally, in glioma (GBM), the PLGA-CMB microbubble complex combined with ultrasound technology overcame the blood-brain barrier, enhanced drug delivery via the cavitation effect, inhibited the PI3K/Akt/mTOR pathway, and induced apoptosis in glioblastoma stem cells (GSCs) (Dong et al., 2022). Collectively, these studies confirm that nanodelivery systems significantly amplify GA’s anticancer effects through optimized drug release, precise targeting, and immunomodulatory functions, offering innovative strategies for treating diverse malignancies.

Despite these advances, GA nanotechnology research still lags behind more established systems such as isorhamnetin in areas of systematic drug evaluation, including long-term stability and *in vivo* pharmacokinetics. Addressing these gaps should be a primary focus to accelerate the clinical translation of GA nanomedicines.

In summary, each nanodelivery platform possesses distinct advantages. Thermosensitive hydrogels enable localized delivery and thermally/chemically triggered release, achieving high local drug concentrations while minimizing systemic toxicity. PLGA nanoparticles combined with UTMD exploit the EPR effect and ultrasound-targeted microbubble disruption to physically breach biological barriers, providing both barrier penetration and spatiotemporal control. Lactoferrin-modified liposomes (LF-lipo) achieve active targeting via LRP-1, which is highly expressed across diverse tumor types, enabling highly selective tumor delivery and suitability for systemic administration to treat metastatic lesions. While local hydrogels demonstrate remarkable efficacy against specific cancers, UTMD remains unparalleled for blood-brain barrier penetration, and photothermal nanoparticles hold immense potential for precise spatiotemporal control. When comprehensively evaluated across three dimensions—broad-spectrum anticancer efficacy, advanced mechanisms such as immunomodulation, and clinical translation feasibility—the LF-lipo strategy demonstrates the most holistic advantages. It has evolved from a simple drug carrier into an intelligent therapeutic system that integrates active targeting, immune microenvironment remodeling, and systemic treatment. This approach aligns closely with contemporary principles of oncological therapy and holds the greatest potential for successfully translating GA into a clinically viable natural anticancer drug, ultimately benefiting patients.

## 5 Future prospects

GA-based antitumor regimens leveraging nano-delivery strategies have demonstrated remarkable advantages (Tables 9, 10). Efforts have been made to design GA nanocarriers for the treatment of malignant tumors, such as nanoparticles targeting the STATs pathway, surface-modified STAT3 inhibitors (e.g., small-molecule JAK2 inhibitors) to synergistically inhibit STAT signaling, and formulations that exploit the EPR effect to enhance local drug accumulation in tumor vasculature. For instance, in nasopharyngeal carcinoma (NPC), such approaches could potentially inhibit metastasis and recurrence by increasing local GA concentration via tumor vascular leakage. Similarly, nanoparticles targeting the PI3K/Akt pathway with surface-modified PI3K inhibitors (e.g., LY294002) could synergistically block PI3K/Akt signaling, thereby enhancing GA’s inhibition of the PI3K/Akt/mTOR pathway, a pathway frequently dysregulated in malignant tumors. These strategies suggest the potential to design GA-based therapeutics with broad-spectrum anticancer efficacy.

Despite significant progress, current nano-delivery technologies—such as pH- or enzyme-responsive carriers and biomimetic nanoparticles—still face challenges. Multicomponent loading may reduce drug-carrying efficiency, while the physiological roles of STAT3 and PI3K pathways raise concerns regarding dual inhibition, such as myelosuppression. Additionally, heterogeneous tumor vasculature can weaken the EPR effect, necessitating integration with localized delivery strategies. Clinical translation is further constrained by production complexity and cost.

Future efforts should focus on developing “smart” delivery platforms with features such as real-time monitoring, controlled release, and synergistic therapeutic functions. For example, nanosystems incorporating photothermal/photodynamic materials or immunoadjuvants could enable multimodal, treatment-integrated therapies, maximizing antitumor efficacy while minimizing systemic toxicity. Beyond oncology and inflammatory diseases, GA’s potential in neurodegenerative disorders (e.g., Alzheimer’s disease), metabolic syndrome, and aging-related pathologies remains largely unexplored. Its antioxidant and antifibrotic properties may prove valuable for organ fibrosis (e.g., lung and liver) and in the regulation of aging-related microenvironments. Furthermore, studies in rare diseases (e.g., hereditary retinopathies) may open new avenues for translational research.

In conclusion, drug development must ultimately serve human health. Research on GA should transcend disciplinary boundaries, integrating nanotechnology, systems biology, and clinical medicine to advance its transformation from a natural product to a clinically actionable therapeutic tool, enabling personalized treatment strategies and maximizing its benefit to human health.
